# ATPe Dynamics in Protozoan Parasites. Adapt or Perish

**DOI:** 10.3390/genes10010016

**Published:** 2018-12-27

**Authors:** Natalia Lauri, Zaher Bazzi, Cora L. Alvarez, María F. Leal Denis, Julieta Schachter, Vanesa Herlax, Mariano A. Ostuni, Pablo J. Schwarzbaum

**Affiliations:** 1 Institute of Biological Chemistry and Physicochemistry (IQUIFIB) “Prof. Alejandro C. Paladini”, Faculty of Pharmacy and Biochemistry, University of Buenos Aires, National Scientific and Technical Research Council (CONICET), Buenos Aires 956, Argentina; natti.lau@gmail.com (N.L.); zaher.bazzi7@gmail.com (Z.B.) cora@qb.ffyb.uba.ar (C.L.A.); mfldenis@qb.ffyb.uba.ar (M.F.L.D.); julischachter@gmail.com (J.S.); 2 Faculty of Pharmacy and Biochemistry, Department of Biological Chemistry, Chair of Biological Chemistry, University of Buenos Aires, Buenos Aires 956, Argentina; 3 Faculty of Exact and Natural Sciences, Department of Biodiversity and Experimental Biology, University of Buenos Aires, Intendente Güiraldes, Buenos Aires 2160, Argentina; 4 Chair of Analytical Chemistry and Physicochemistry, Faculty of Pharmacy and Biochemistry, Department of Analytical Chemistry, University of Buenos Aires, Buenos Aires 956, Argentina; 5 Biochemistry Research Institute of La Plata (INIBIOLP) “Prof. Dr. Rodolfo R. Brenner”, Faculty of Medical Sciences, National University of La Plata, National Scientific and Technical Research Council, Av. 60 y Av., La Plata 120, Argentina; vherlax@med.unlp.edu.ar; 6 National University of La Plata, Faculty of Medical Sciences, Av. 60 y Av., La Plata 120, Argentina; 7 UMR-S1134, Integrated Biology of Red Blood Cells, INSERM, Paris Diderot University, Sorbonne Paris Cité, University of La Réunion, University of Antilles, F-75015 Paris, France; mariano.ostuni@inserm.fr; 8 National Institute of Blood Transfusion (INTS), Laboratory of Excellence GR-Ex, F-75015 Paris, France

**Keywords:** parasite, membrane proteins, host–parasite interaction, transport, pathogenesis, evolution

## Abstract

In most animals, transient increases of extracellular ATP (ATPe) are used for physiological signaling or as a danger signal in pathological conditions. ATPe dynamics are controlled by ATP release from viable cells and cell lysis, ATPe degradation and interconversion by ecto-nucleotidases, and interaction of ATPe and byproducts with cell surface purinergic receptors and purine salvage mechanisms. Infection by protozoan parasites may alter at least one of the mechanisms controlling ATPe concentration. Protozoan parasites display their own set of proteins directly altering ATPe dynamics, or control the activity of host proteins. Parasite dependent activation of ATPe conduits of the host may promote infection and systemic responses that are beneficial or detrimental to the parasite. For instance, activation of organic solute permeability at the host membrane can support the elevated metabolism of the parasite. On the other hand ecto-nucleotidases of protozoan parasites, by promoting ATPe degradation and purine/pyrimidine salvage, may be involved in parasite growth, infectivity, and virulence. In this review, we will describe the complex dynamics of ATPe regulation in the context of protozoan parasite–host interactions. Particular focus will be given to features of parasite membrane proteins strongly controlling ATPe dynamics. This includes evolutionary, genetic and cellular mechanisms, as well as structural-functional relationships.

## 1. Introduction

Adenosine 5′ triphosphate, ATP, is usually described as an essential energy source for cells. ATP hydrolysis provides the energy required for many chemical reactions of metabolism, acts as a precursor of a number of essential enzyme cofactors, such as nicotinamide adenine dinucleotide (NAD) and coenzyme A, and is the source of the phosphoryl group in most kinase-mediated phosphorylation reactions [1,2]. ATP is also used to synthetize cyclic adenosine monophosphate (cAMP), a major second messenger involved in the regulation of numerous cellular processes.

A less known function for ATP is to act as an extracellular signaling molecule, allowing cells, tissues, and organisms to communicate [3]. In addition, several studies during the last 30 years show that extracellular ATP (ATPe) is involved in modulating a vast array of cellular functions, such as neurotransmission, secretion of pro-inflammatory mediators, apoptosis, chemotaxis, cell differentiation, and vasodilatation [3].

In cells and organisms, ATPe is subjected to a sophisticated system of regulation. At least four processes are required to understand ATPe regulation at the cellular level: (1) ATPe, and/or some of its breakdown products can interact with specific purinergic receptors (P receptors), thus triggering ionotropic and/or metabotropic cellular responses [3]; (2) intracellular ATP can be released by cell rupture (cytolysis) or by regulated mechanisms involving membrane transport proteins [4,5]; (3) ATPe can be metabolized by specific membrane bound enzymes collectively called ecto-nucleotidases [6,7]; (4) full dephosphorylation of ATPe and other extracellular nucleotides leads to accumulation of nucleosides, which promotes uptake of these molecules by nucleoside salvage mechanisms [8]. The mechanisms involved in ATPe regulation are usually cell type and species specific, and may often change according to the pathophysiological context [3]. The picture turns even more complex during parasite infection of targeted hosts [9,10].

At the evolutionary level, a continuous crosstalk exists between the parasite and the host leading to adaptive changes allowing parasites to invade, grow and survive inside the host, in an attempt to subvert host defense mechanisms to ensure development of the infection [11].

Such changes may include adapting host molecules, metabolic pathways and transporters for their specific needs, and remodeling host membranes to avoid clearance. Moreover, parasites undergo successive stages during their life cycle and may alternate between different hosts and tissues, resulting in diverse combinations of host–parasite interactions. At the physiological level, on the other hand, molecular, cellular and systemic adjustments can be relatively fast.

In humans, while transient increases of ATPe are involved in physiological signaling, strong increases of ATPe concentration can occur in seconds, and serve as a danger signal in pathological conditions, triggering a complex immunological and inflammatory response aimed at recognizing and controlling intracellular pathogens [10,12]. In this context, infection by protozoan parasites has been shown to alter host ATPe dynamics, by promoting changes in at least one of the above-mentioned mechanisms controlling ATPe concentration. Protozoans may display their own set of proteins directly altering ATPe dynamics or control the activity of host proteins. Parasite-dependent activation of ATPe conduits [13,14] of the host may promote infection and systemic responses that are beneficial or detrimental to the parasite [15,16,17], while protozoan ecto-nucleotidases can affect parasite growth, infectivity and virulence [18,19]. Both actions on ATP efflux and ATPe hydrolysis mediate cellular responses by altering the availability of nucleotide ligands involved in purinergic signaling of the host.

In this review, a special focus is given to protozoan proteins involved in mechanistic interactions that regulate the homeostasis of ATPe and byproducts as well as the consequences of this regulation for the parasite and the host. When information on specific protozoan proteins is scarce, a brief description of related groups was included.

## 2. Purinergic Signaling

Signaling by P receptors appeared early in evolution as a form of chemical intercellular communication, possibly with the first unicellular eukaryotic cells [2]. Further evolution in multicellular organisms resulted in the development of multiple classes of P receptors. Cloning of P receptors [20] allowed for the production of polyclonal antisera for immunohistochemical studies of their expression and distribution, showing that most receptors were located on the cell surface, though in a few cases intracellular immunostaining was observed (see [21]).

The P indicates that these receptors can be activated by purine or pyrimidine nucleotides (P2) or by the nucleoside adenosine (P1). P2 receptors are separated into two major subfamilies, P2X and P2Y. Several comprehensive reviews summarize the accumulated knowledge on these proteins [3,22]. Cloned subtypes include seven P2X receptors (P2X1–7), eight P2Y receptors (P2Y1, P2Y2, P2Y4, P2Y6, P2Y11–14) and four P1 receptors (A1, A2A, A2B, and A3) [23,24]. P2Y and P1 receptors are metabotropic G-protein coupled receptors. Binding of di- or trinucleotides (P2Y) or adenosine (P1) triggers second messengers which in turn will activate specific protein kinases coupled to downstream signaling mechanisms [3].

As described in Section 4, extracellular metabolism of nucleotides facilitated by membrane bound ecto-nucleotidases will enable the accumulation of several purine and pyrimidine di- and trinucleotides and nucleosides. The effective concentration of these molecules at the cell surface will produce metabolic and physiological responses by interacting with specific subtypes of P2Y and P1 receptors [25,26].

In contrast to P2Y and P1, P2X receptors are ionic channels activated only by ATPe, its only natural ligand [22]. In this section, we will focus mainly on P2X receptors, since these are assumedly the first to appear in protozoa, according to the limited but consistent genomic, pharmacological and biophysical information [2,27]. Most P2X channels are cation-selective and discriminate poorly among Na^+^, K^+^, and Ca^2+^, the main diffusible cations of eukaryotes. Transmembrane transport of these ions is an important signaling mechanism, because it may alter the plasma membrane potential as well as local ion concentrations [20].

Transcripts and/or proteins of P2X subunits have been found in most mammalian tissues and are being increasingly discovered in several non-vertebrate species [28]. They exhibit a common topology with two transmembrane domains, a large ATPe binding loop, and intracellular N-and C-termini of variable lengths [29]. The ecto-domain is glycosylated and includes ten Cys residues conserved among all vertebrate receptors, bound in five disulphide bridges contributing to the tertiary structure of the receptor [20].

P2X receptors are able to form homo-and heterotrimeric complexes [30,31] around a central pore [32,33], and may be regulated by post-translational glycosylation and phosphorylation [34]. The trimeric structure was confirmed by atomic force microscopy, electron microscopy, single particle analysis, and by crystallization of the P2X4 subtype from zebrafish [28], which agrees well with ion conductance recordings suggesting that three molecules of ATP are required for channel gating [35,36].

### 2.1. P2X Receptors. Evolution and Early Appearance in Protozoa

As mentioned earlier, seven mammalian P2X cDNAs have been cloned [28]. Based on sequence identity and pharmacological profile, most receptors of vertebrate species seem to be orthologs of these receptors but low sequence homology has made difficult to determine potential homologues in non-vertebrate organisms. Currently, no prokaryotic P2X receptor has been identified, suggesting that structurally different ATP receptors evolved in bacteria [28,37]. P2X receptors appear to be absent in several invertebrates such as *Caenorhabditis elegans*, *Drosophila melanogaster*, *Anopheles gambiae*, and *Apis mellifera* [28], although extracellular nucleotides induce various cellular responses in tissues of these animals [38]. On the other hand, P2X-like receptors were identified in the unicellular algae *Ostreococus tauri* [39]. In vascular plants, although genomic sequence-based searches for canonical P2 receptors failed to detect candidate ATP receptors [40], ATPe and other extracellular nucleotides are able to trigger several cellular and systemic responses [41]. Recently, a lectin receptor kinase was found in *Arabidopsis thaliana*, which binds ATPe with high affinity and is required for ATPe-induced intracellular Ca^2+^ increase [40].

Alignment of P2X proteins from a diverse range of species suggests that the P2X1-7 receptor subtypes resulted from a lineage-specific gene expansion [42]. Similar but independent lineage specific expansions of P2X receptor subtypes may have occurred in amoeba *Dicthiosthelium discoideum* and algae *O. tauri*. *D. discoideum* possesses five P2X-like sequences, while *O. tauri* exhibits an identified P2X receptor together with three further open reading frames encoding proteins assumed to be distantly related to P2X receptors [43].

The protein sequences, kinetics and pharmacology of protozoan P2X-like receptors do not seem to correspond to a specific P2X receptor subtype [39,43,44,45], which is not surprising considering that development of the seven mammalian P2X receptor genes appeared to be a relatively recent phenomenon occurring after the branching between vertebrates and invertebrates [28]. Sensing nucleotides has adaptive value for protozoans, since they control a wide range of different processes like cilia beating, swimming, and chemotaxia in *Paramecium* [46] and *Tetrahymena* [47]; induction of parasitosis in *Trypanosoma cruzi* [48]; vacuole contraction in *Amoeba proteus* [49]; changes in the cytoskeletal organisation in *Physarum* [50]; and phagocytosis in *Dicthiosthelium* [42].

#### 2.1.1. A Brief Tale of Three Protozoans

##### *Monosiga brevicollis* 

Choanoflagellates are among the closest unicellular relatives of animals and provide important insights into the origin and diversity of animal phyla. These protozoa constitute the major component of aquatic microbial foodwebs [27,51]. The genome of the marine choanoflagellate, *Monosiga brevicollis*, has been sequenced in 2008 [52]. Because of horizontal gene transfer, >400 genes are likely derived from algae and prokaryotes, accounting for ≈4% of *Monosiga* nuclear genome [52]. Interestingly, among the 42 Mb genome containing approximately 9200 predicted protein coding genes, a P2X-like receptor (named MBP2X) was detected. When expressed in human embryonic kidney cells (HEK-293), MBP2X forms a functional ATP activated ion channel, which may be implicated in flagella driven swimming or feeding of this organism [27], though a biophysical/phamacological characterization remains to be performed.

##### *Dictyostelium discoideum* 

A P2X-like gene encoding protein DDB0168616 appeared in *D. discoideum* [43], an amoeboid species whose genome was fully sequenced in 2005 [53].

Expression of a humanized version of this cDNA in HEK-293 cells showed that this gene (denoted as *D. discoideum* p2xA) encoded a membrane ion channel (DdP2X) activated by micromolar concentrations of ATPe as well as slow-degradable ATP analogues [43]. In HEK-293 cells, ATPe elicited whole-cell currents in a dose-dependent manner, with kinetic properties resembling human P2X2 or P2X4 receptors, although typical purinergic blockers did not inhibit the response [43]. On the other hand, site-directed mutagenesis revealed partial conservation of structure–function relations with P2X receptors of higher organisms. For example, as in mammalian P2X receptors, two lysine residues in the receptor ecto-domain contribute to ATP binding and a C-terminus YXXXK motif is involved in receptor stabilization at the plasma membrane [33,43]. Moreover, expression of the recombinant receptor in mammalian cells suggests conservation of trimer formation—similar to homologs of vertebrates—by DdP2X [43]. A green fluorescent protein (GFP)-tagged version of DdP2X was localized to the intracellular membranes of *D. discoideum*, mostly in the contractile vacuole, a specialized organelle in osmoregulation. Thus, DdP2X functions on intracellular organelles, a feature observed in mammal cells after constitutive or ligand-activated endocytosis of surface receptors [21]. DdP2X is oriented with the ATP binding site facing the vacuole lumen, thereby being in principle able to sense changes in luminal ATP. Targeted disruption of DdP2X resulted in cells being unable to regulate cell volume when challenged by hypotonicity [43]. A similar P2X dependent regulation of cell volume can be found in human red blood cells (RBCs), where surface located P2X receptors subtypes control cell volume by modulating the net flux of cations [54,55].

Interestingly, although DdP2X is strictly intracellular, *D. discoideum* also exhibits purinergic signaling induced by extracellular purine nucleotides [42], i.e, exposure to ATPe or ADPe trigger changes in intracellular Ca^2+^ content, which are comparable to the role for P2 receptors in vertebrate cells [56]. More recently, Sivaramakrishnan and Fountain (2015, [57]) showed that *Dictyostelium* uses ATPe to regulate cell volume. In most eukaryotic cells from multicellular organisms, swelling leads to intracellular ATP release. The accumulated ATPe, and/or its metabolic byproducts, interacts with P receptors mediating a decrease of cell volume [25,26]. Although a similar response sequence is observed in *D. discoideum* challenged by hypotonicity, the genome of this protozoan yields no information suggesting a putative cell surface P2 receptor [57].

##### *Plasmodium falciparum*, a Parasitic Protozoon

*Plasmodium falciparum* is the most dangerous etiological agent of human malaria [58]. After entering into the human body, *P. falciparum* first undergoes a developmental stage in the liver before invading RBCs, where the parasite grows and matures [59]. Classical antimalarial therapy is directed against the intraerythrocytic stage [60], which produces all symptoms of the disease [59]. The merozoite (parasite invading phenotype) invades the RBC and grows and replicates within the parasitophorous vacuole (PV), undergoing development through well-characterized stages of ring, trophozoite, and schizont. Subsequently, the infected RBC ruptures, releasing new merozoites that in turn infect more RBCs [59].

Sequencing of the parasite genome [61] revealed approximately 60% of the predicted genes not found in other organisms, thus hampering the search for homologous genes. This genomic diversity is in part due to the extreme evolutionary divergence of the phylum Apicomplexa, but also to species-specific features, with proteins in *P. falciparum* differing from those of the other closely related Plasmodia species [62].

**Figure 1:**
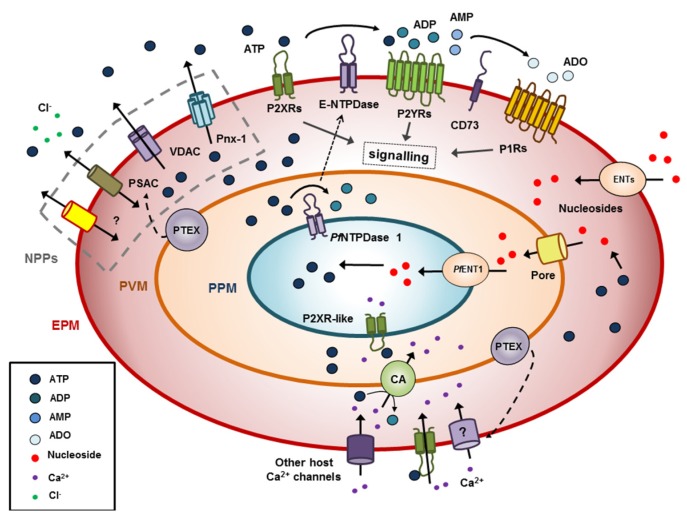
Extracellular ATP (ATPe) dynamics of *Plasmodium falciparum* infected erythrocytes. The parasite induces a general increase of solute permeability at the host cell membrane (EPM; erythrocyte plasma membrane), via new permeability pathways (NPPs), which includes an increase of ATP efflux mediated by several proteins like pannexin 1 (Pnx-1), anionic channels such as voltage-dependent anion channel (VDAC) coupled to partner proteins, generic solute pores as plasmodial surface anion channel (PSAC) and novel parasite proteins yet to be discovered. ATPe hydrolysis by red blood cells (RBCs) E-NTPDases (ecto-nucleoside triphosphate diphosphohydrolases) is extremely low but strongly increases under infection, though participation of parasite NTPDases at the host membrane remains to be proven. Still, ATPe and its metabolic byproducts should be able to activate functional P1 and P2 receptors of the host, triggering intracellular signaling. In uninfected RBCs, part of this signaling was shown to affect ATP efflux. On the other hand, accumulation of nucleosides (due to hydrolase activities of E-NTPDases and CD73) will promote their uptake by host equilibrative nucleotide transporters (ENT). Nucleosides will then transverse both unspecific vacuolar pores (Pore) of the parasitophorous vacuole membrane (PVM) and PfENT1 of the parasite plasma membrane (PPM) to gain access to the parasite. Nucleosides will then serve as substrates in the parasite pathway for de novo synthesis of nucleotides. The parasite traslocon PTEX was shown to transport parasite protein(s) to the host membrane, EPM, upregulating Ca^2+^ uptake. Once in the RBC cytoplasm, Ca^2+^ may be transported through the parasitophorous vacuole (PV) by a calcium ATPase transporter, potentially reaching the parasite cytoplasm through a P2X-like receptor conductance. On the other hand PTEX may be involved in the translocation of parasite proteins to the NPPs, as e.g., PSAC.

Plasmodia spp. employ unique proteins involved in highly specialized processes, such as passage through blood vessels, evasion of the host immune system, and invasion and development within host cells [63]. To facilitate intracellular growth, *P. falciparum* hijacks host organelles for nutrient uptake, adapts the parasitophorous vacuole membrane (PVM) and the host membrane by exporting many proteins, and increasing erythrocyte plasma membrane (EPM) permeability to a wide variety of organic and inorganic solutes [64,65,66] (see Figure 1).

The challenge for the parasite is to widely and unspecifically raise membrane solute traffic but excluding Na^+^, whose permeability should be kept low to avoid RBC lysis. Different proteins were proposed to be involved in these new permeability pathways (NPPs) induced by parasite infection. Among them, in the last years new experimental data support a crucial role for the plasmodial surface anion channel (PSAC) [66,67,68], associated with different proteins from Clag and RhopH families [67]. However, an alternative, non-exclusive hypothesis states that infection activates host proteins or protein complexes, which were expressed but inactive or weakly active in the uninfected RBC [69].

Amongst inorganic ions with increased permeability, Ca^2+^ uptake proved to be the most critical for intracellular parasite performance [63], since the *P. falciparum* expresses multiple Ca^2+^ effector proteins like calmodulin, Ca^2+^-dependent protein kinases, phosphatases such as calcineurin, and other Ca^2+^-binding proteins [70]. Early works have shown that both parasite invasion and the propagation of in vivo infected RBCs cultures required the presence of extracellular calcium [71,72]. At rest, *P. falciparum*, as most eukaryotic cells, exhibits submicromolar intracellular Ca^2+^ concentration [63]. However, intracellular Ca^2+^ concentration is highly increased when isolated parasites are treated with ATP in the presence of extracellular Ca^2+^, thus suggesting an activation of Ca^2+^ influx. Moreover, experiments in Ca^2+^ free medium abrogated the response, indicating that the parasite possesses an ATP-gated calcium channel [15], a form of primitive P2X receptor (Figure 1). Similar responses were obtained with *Plasmodium yoelii* and *Plasmodium berghei* (two species infecting rodents) [73]. Unlike in the case of *D. discoideum* DdP2X receptor, a set of blockers (IP5I, PPADS, KN-62, TNP-ATP, and Suramin) known to act on cloned vertebrates P receptors significantly inhibited the rise in ATPe-dependent intracellular Ca^2+^ concentration. Surprisingly, extracellular UTP also induced intracellular Ca^2+^ deregulation in the parasite, a featured not observed in P2X1-7 receptors [15].

Several lines of evidence suggest that such P2X-like receptor of *P. falciparum* can be important for parasite invasion to RBCs: (1) ATPe and Ca^2+^, but not other divalent cations triggered parasite invasion [15,63] and ATPe-dependent intracellular Ca^2+^ concentration increase is required to ensure the progress of the parasite life cycle [70]. (2) Under in vitro culture of RBCs with parasites, removing endogenous ATPe by apyrase or pre-incubation with P2X blockers, drastically impairs parasite invasion [15]. Interestingly in this context, exogenous ATP does not improve the efficiency of RBC infection, suggesting that the putative P2X receptor should exhibit a relative high affinity for ATPe. Alternatively, ATP affinity could be moderate, but receptor activation is possible in light of experiments showing an increased ATP efflux from infected RBCs throughout the parasite life cycle, which correlated with elevated ATPe concentrations in infected RBCs cultures with *P. falciparum* [14,74] and plasma samples from humans [75]. Once inside the RBC, *P. falciparum* is enclosed in a highly permeable PVM, and exposed to the RBC cytoplasm containing a very low intracellular Ca^2+^ concentration (<100 nM), which is much lower than in other cell types, and at least 3 orders lower than the millimolar Ca^2+^ concentration of the plasma. A very low Ca^2+^ concentration environment set by the host is incompatible with parasite functions and survival and may hamper a potential role of parasite P2X-like receptor in modulating ATP-dependent calcium influx [76]. In line with this notion, Gazarini et al. [76] nicely demonstrated that *P. falciparum* and *Plasmodium chabaudi* exhibit an active mechanism for Ca^2+^ accumulation in the immediate parasite microenvironment, with concentrations of the cation amounting to 40 µM. This is much lower than plasma concentrations of the cation, but nevertheless 100–1000-fold higher than that inside the parasite and in the RBC cytosol [77,78]. Thus, a Ca^2+^ gradient does exist between the relatively low intracellular Ca^2+^ concentration of the parasite inside the PV and its external milieu, making a P2X-like receptor dependent Ca^2+^ uptake in the parasite functionally relevant (Figure 1).

In addition, *P. falciparum* can activate Ca^2+^ influx of the RBC host. Early ^45^Ca^2+^ flux studies indicated that net Ca^2+^ entry into infected RBCs was approximately 20 times faster than into uninfected cells [79], which agrees well with the reported elevated Ca^2+^ conductance of patched membranes from infected RBCs [80]. This increased accumulation rate could not be explained by inhibition of the Ca^2+^ pump, a cell membrane ATPase that keeps the Ca^2+^ of uninfected RBCs very low. More recent pharmacological studies suggested that the observed elevated Ca^2+^ permeability was mediated by a new mechanism activated in the infected host cell [71]. I.e., when RBCs were infected with a parasite knocked down for PTEX (a translocon for export of parasite proteins into the host cell), Ca^2+^ uptake was highly reduced. Moreover, a cell-based high-throughput screen for specific Ca^2+^ transport inhibitors confirmed a protein-mediated uptake mechanism on infected cells distinct from the low capacity Ca^2+^ carrier of RBCs [79] (Figure 1).

### 2.2. P receptors of the Host and Parasite Infection

Host organisms have evolved several mechanisms to control infection, while protozoa parasites display several adaptations to evade host defense. P receptors of the host are often involved in this fight [9,10,81].

*Toxoplasma* infects practically all mammalian cells [82]. Although most infections are asymptomatic, they can cause serious problems when the immune system is compromised as with pregnant women, AIDS patients or during congenital transmission [83,84]. Similarly to Plasmodia, invasion results in the establishment of a subcellular PV. Success of the parasite depends on its ability to rapidly reproduce inside the PV and then for its progeny to escape from the membranes of the vacuole and that of the host cell, so as to invade more cells [85,86]. Following infection, the parasite inhibits the fusion between lysosomes and the PV, maintaining the extravacuolar medium at a neutral pH, thus allowing parasite survival [82]. However, ATPe ligation of P2X7 receptors restores that fusion, acidifying the PV and leading to elimination of *Toxoplasma gondii* in macrophages [87,88]. In addition, activation of P2X7 receptors in *Toxoplasma* infected macrophages increases the production of reactive oxygen species [87,89] and induces apoptosis [88], another two host defense responses.

To the extent that host strategies require an efficient, fully functional P2X7 receptor, polymorphisms at the *P2X7* gene were shown to influence the susceptibility to toxoplasmosis in certain human populations [90]. In particular, macrophages from people with the 1513C loss-of-function single nucleotide polymorphism are less effective to kill *T. gondii* after treatment with ATP than macrophages from people with the 1315C wild-type allele [88]. Thus P2X7 receptor activation of specific polymorphic variants may influence the outcome of infection, no matter the route of transmission or parasite genotype, which varies considerably worldwide [90]. In line, macrophages from P2X7 receptor knock-out mice are not able to eliminate the parasite as successfully as macrophages from wild-type mice [88].

Parasites may counteract P2X7 receptor mediated attack by secreting ecto-nucleotidases of the E-NTPDase (ecto-nucleoside triphosphate diphosphohydrolase) family (see Section 4) before and after host cell entry [91,92], to promote ATPe hydrolysis and therefore a decrease in P2X7 receptor activation. As analyzed in Section 4, virulent *T. gondii* strains express the enzyme TgNTPDase 1, which possess typical signal peptides for secretion and displays high ATPase activity [92,93]. At least theoretically, TgNTPDase1 should be able to facilitate a decrease of ATPe concentration, thus lowering P2X7 receptor activity and dampening the inflammatory response. While this reasoning is still speculative, compelling evidence has revealed an important role for E-NTPDases in P2X7 receptor activity by controlling the effective ATP concentration at the cell surface, and the availability of ATPe during infection-induced inflammation [94].

In addition to downregulation of P2X7 receptor activity, an increase ATPe hydrolysis by TgNTPDase1 may have consequences on downstream purinergic signaling, by facilitating the accumulation of extracellular AMP, and further degradation to adenosine by host ecto-5′-nucleotidase. Elevated adenosine, in turn, may activate host P1 receptors. In contrast to ATPe, adenosine has anti-inflammatory and immunosuppressive effects on immune cells [95,96]. Although neutrophils are recruited during *T. gondii* infection [97], activation of P1-A2A receptors on these cells by extracellular adenosine was associated with inhibition of activation and migration [98]. Moreover, infection with *T. gondii* is lethal in mice deficient in 5′-ecto-nucleotidase, unless a potent P1 agonist is administrated [99], and mice deficient in the adenosine receptor P1-A2A are more susceptible to infection than wild type counterparts. Thus, production of adenosine by host 5′-ecto-nucleotidase, probably facilitated by TgNTPDase 1 degradation of ATPe, leads to increased susceptibility of mice to *T. gondii* infection. Several strategies of *Leishmania* are different from those of *Toxoplasma*. *Leishmania* species are the ethiological agents of leishmaniases. Parasites are transmitted through the bites of more than 90 different species of phebotomine sandflies, and have developed several strategies to survive within the host by evading the immune response.

After invading macrophages by phagocytosis, *Leishmania* can survive within an acid pH environment inside the phagolysosomes. In addition, *Leishmania* interferes with the production of reactive oxygen and nitrogen species by phagocytes [100]. Macrophages infected with *Leishmania amazonensis* exhibit higher expression of P2X7 receptors than noninfected cells [101]. ATP treatment reduced parasite load [101,102] though not in macrophages isolated from P2X7 knockout mice [102].

In addition to P2X7 receptor modulation, infection with *L. amazonensis* up-regulates the expression of P2Y2 and P2Y4 metabotropic receptors, which can be activated by either ATP or UTP [103]. This is again a host antimicrobial response, since UTP decreases parasite load [104]. P2Y2,4 receptors signaling involves intracellular Ca^2+^ mobilization, probably inducing apoptosis of infected cells as well as production of reactive oxygen and nitrogen species [104].

As in the case of *Toxoplasma* infection, effects of adenosine on *Leishmania* parasitism were also reported [105]. Patients with visceral leishmaniasis present higher concentrations of adenosine in serum than healthy people [106]. For mice infected with *Leishmania braziliensis*, administration of adenosine increased lesion development and tissue parasitism [107], while blockage of P1-A2B receptors decreased lesion size and parasitic load [107]. In a murine model of *Leishmania infantum* infection, P1-A2A receptor signaling reduced activation and migration of neutrophils, allowing the establishment of infection [108].

An additional mechanism for regulation of host P2 receptors could involves desensitization, which occurs in P2X1, P2X3, and P2Y1 receptors [21] by long exposure to their natural ligands. Considering that parasite infection alters the kinetic profiles of extracellular nucleotide accumulation, it may well regulate P2 receptors activity by affecting P2 desensitization. For instance, platelets activation of P2Y1 by extracellular ADP highly mediates aggregation [109]. During *P. falciparum* infection, strong ATP efflux of infected RBCs, together with a highly increased ATPe hydrolysis by upregulated ecto-nucleotidases [14], should promote a strong enhancement of ADPe in the blood stream. Under these conditions, platelets P2Y1 receptor may desensitize, and thus undergo a refractory state characterized by the inability to aggregate or change shape in response to extracellular ADP. Changes in ATPe concentration may also affect desensitization of P2X1 receptor, causing abnormalities in blood coagulation [109]. In patients with malaria, both hyperaggregation and defective aggregation were described [110], though the contribution of extracellular nucleotides in this regulation needs to be confirmed.

## 3. Transport of ATP

In cells and organisms multiple compartments exist from where ATP can be transported, depending on the effective concentration, the available transmembrane ATP gradients (the driving force for conductive ATP flux) and the capacity and kinetics of several proteins allowing ATP permeation.

Different cells from unicellular and multicellular organisms release ATP and other nucleotides (ADP, UTP, and UDP) and adenosine during mechanical injury, necrosis, apoptosis, as well as under various mechanical and chemical stimuli, such as shear stress, hypotonic swelling, stretching, and different Ca^2+^-mobilizing agonists [111,112,113,114].

Most non-lytic mechanisms of ATP release were studied in mammals, where conduits for regulated vesicular exocytosis or conductive nucleotide release via ion channels and transporters were identified [54,55,113]. In principle, two main classes of plasma membrane channels have been associated with ATP conductive activity: (1) Cl^−^ channels such as the calcium homeostasis modulator 1 (CALHM1; [5]) channel, maxi-anion channels, volume-regulated anion channels (VRAC, also known as volume-sensitive outwardly rectifying anion channel), and tweety [113,115,116]; and (2) pore forming connexins and pannexin-1 (pnx-1). In addition, P2X7 receptor, alone or in association with pnx-1, is known to progressively generate a large membrane pore that allows ATP permeation [117]. Other conduits such as the voltage-dependent anion channel (VDAC) were shown to interact with partner proteins, like the 18-kDa-tranlocator protein (TSPO) and the adenine nucleotide transporter (ANT). Thus, it soon become established that, although most Cl^-^ channels are able to permeate ATP to different extents [118,119], ATP transport may require these channels to operate as part of multimolecular complex systems [120].

Evidence of ATP transport conduits of protozoa is scarce. In *D. discoideum*, ATP is released following a hyposmotic challenge. Pharmacological evidence suggests an exocytotic mechanism, but the implicated mechanisms were not identified [57]. *D. discoideum* appears to harbour an ATP translocation mechanism to mediate ATP accumulation within the vacuole lumen. However, this is not the source of the observed ATP release, since knockout cells with impaired contractile vacuole fusion exhibited no inhibition of ATPe accumulation [57]. Irrespective of the mechanism, the resulting osmotically induced accumulation of ATPe could signal auto/paracrinally through a putative cell surface P2-like receptor [15].

Microsporidians are eukaryotic unicellular organisms living as obligate intracellular parasites of vertebrates and invertebrates. Initially thought to be protozoans, recent molecular studies indicate that the phylum Microspora should be more closely related to Fungi kingdom [121]. Recently, microsporidians have been reported to parasite humans, especially those immunologically compromised [122]. These organisms have undergone massive gene loss in the transition to obligate intracellular parasitism. Some of these genes encode proteins involved in purine and pyrimidine biosynthesis, and the oxidative synthesis of ATP. On the other hand, glycolysis is highly down regulated [123]. Thus, replicating parasites must import ATP from the host cell, using some kind of transport system.

Microsporidian genome shows genes encoding for nucleotide transporters (NTTs) capable of transporting ATP [124]. Three NTTs are located to the plasma membrane, while one is localized to the mitosome, a remnant mitochondrial organelle [125]. Genome sequences of several microsporidian species contain one or more of these transporters, probably acquired by lateral gene transfer from intracellular bacterial pathogens [124], enabling early microsporidians to take up host ATPe for their growth and biosynthesis processes. Expression in *Escherichia coli* suggests that NTTs display high affinity for ATP, while binding competition assays show that other nucleotides can be transported as well. Clearly in this case, ATPe of microsporidian is the cytosolic ATP of the host. Signaling of ATP might not be important in this case, but the energetic need for nucleotide salvage.

A similar strategy is used by protists living as endosymbionts of amoeba and paramecia, although ATP transport involves an ATP/ADP exchange mechanism [126].

Genes coding for these exchangers were found in several species of *Rickettsiae* and *Chlamydiae*, promoting import of host ATP through the prokaryotic cell membrane, which is otherwise impermeable for relatively large anionic nucleotides, in exchange for ADP export into the host cytosol [126]. ATP-ADP exchange was also verified when expressed in a heterologous bacterial system. 

Although in mammals such an ATP/ADP exchanger occurs exclusively in the inner mitochondrial membrane, ANT has been observed in the plasma membranes of RBCs [120,127]. As we mentioned previously, ANT may physically associate with VDAC and other proteins at the RBC cell membrane, to form a conduit capable of exporting ATP to the extracellular milieu [120].

In Plasmodia, ATP and other organic anions were proposed to be transported by the NPPs, though the molecular identity of this pathway is a matter of debate. However, it was recently reported that drug ligands modulating VDAC activity were able to induce ATP release from RBCs [120,128], and that *P. falciparum* infection induced changes in the affinity of RBC membranes for these drug ligands [69].

In protozoan parasites, use of host ATP is qualitatively different to microsporidians and protists parasites, in that ATP and other nucleotides are first dephosphorylated by ecto-nucleotidases to nucleosides, purines, and pyrimidines. Specific nucleoside transporters allow uptake of these compounds into the parasite, which can then be used to resynthesize ATP and nucleic acids (see Section 5).

In many cells and organisms, hypoxia and anoxia constitute potential stimuli promoting regulated ATP release by vesicular and conductive mechanisms [129,130,131,132]. In human RBCs, hypoxia-induced ATP release is mediated by pnx-1 [133], although other stimuli can activate different ATP conduits in these cells [54,55,128].

Interestingly in this context, *Leishmania* promastigotes (the flagellated phenotype) were shown to induce non-lytic release of intracellular ATP, but not of other nucleotides, when challenged by anaerobiosis [134]. The accumulated ATPe may have various fates. On the one hand, *Leishmania* or host derived ATPe might activate parasite killing by activation of P2X7 receptors of macrophages [101]. Alternatively, ATPe may be degraded by *Leishmania* NTPDases, as part of a mechanism for purine salvage ([135]; see Section 5).

In addition, ATPe may participate in the phosphorylation of extracellular proteins, affecting the interaction of the parasite with the host’s immune system. Accordingly, exogenous ATP was shown to phosphorylate added histone and several parasite surface proteins, including tubulin [136].

## 4. Ecto-Nucleotidases

Ecto-nucleotidases are plasma membrane-associated nucleotidases that usually display their active site to the extracellular space, although cleaved and soluble extracellular isoforms may occur [137]. The currently known ecto-nucleotidases include members of the E-NTPDase family (ecto-nucleoside triphosphate diphosphohydrolases), hydrolyzing extracellular nucleoside 5′ di-and triphosphates, E-NPP (ecto-nucleotide pyrophosphatase/phosphodiesterases), which can hydrolyze pyrophosphate 5′-monodiester bonds in ATP and dinucleoside polyphosphates; ecto-alkaline phosphatases, acting as non-specific phosphomonoesterases; and ecto-5′-nucleotidases, also called CD73, which hydrolyse only nucleosides monophosphates [6]. Extracellular adenosine can thereafter be either inactivated by cell surface associated adenosine deaminase (ADA) and purine nucleoside phosphorylase (PNP; [114,138,139]) or transported into the cell by equilibrative or Na^+^-dependent nucleoside transporters in order to replenish the intracellular nucleotide pool, as discussed in Section 5 [140,141].

In different organisms, the relative contribution of different ecto-nucleotidases to the regulation of extracellular nucleotides depends not only on the availability and preference of substrates, but also on their cell and tissue distribution [34]. Hydrolysis of ATPe and other extracellular di- and trinucleotides is mainly promoted by different E-NTPDase subtypes, which are ubiquitously expressed in eukaryotes [6,142]. To date there are eight members of this family. NTPDases 1, 2, 3, and 8 are expressed as cell surface locate enzymes, with the catalytic site facing the extracellular milieu. NTPDases 5 and 6 are intracellular, but can be proteolitically cleaved and secreted, while NTDPases 4 and 7 are intracellular with the active site facing the lumen of organelles [6,114,137].

All E-NTPDases are highly glycosylated, integral membrane proteins, which require intracellular Ca^2+^ or Mg^2+^ for enzyme activity [114,143]. All members share five highly conserved sequences of amino acids called apyrase conserved regions (ACRs), which are essential for enzyme function [137,144,145,146,147], and may exist either as monomers or homo-oligomers [6,137,147]. Site-directed mutagenesis studies of conserved amino acid residues in ACRs regions show that, depending on the amino acid being altered, site mutations resulted in inactivation or activation of enzyme activity [147,148], while significant changes in the kinetic features are possible with only a few changes in the amino acid sequence of the ACRs [148].

The different subtypes of E-NTPDases differ in their preference for nucleotide 5′-tri-and nucleotide 5′-diphosphates, displaying broad substrate specificity towards different purine and pyrimidine di- and trinucleotides, with Km values ranging from 50 to 200 µM [7]. These values should be taken with caution, since the catalytic properties and nucleotide preferences obtained in vitro with enzymes purified to different extents and with different detergents may not match the in vivo kinetic features of membrane bound enzymes. In COS7 cells transfected with cDNA coding for human and murine E-NTPDases 1, 2, 3, and 8, most apparent Km values for these enzymes were in the low micromolar range [143].

For each system and a specific metabolic status, the rate of nucleotide to nucleoside conversion is relevant, since it determines the effective time a given nucleotide is available to interact with specific P receptors before being locally hydrolyzed by ecto-nucleotidases. In several cell types [149,150], E-NTPDases may act in concert with the 5′-ecto-nucleotidase to achieve the sequential and complete dephosphorylation of ATPe to adenosine. The 1-8 classification of the E-NTPDase family was mainly derived from studies on animal tissues [151], but—as we will see below—evolutionary earlier forms of these enzymes may differ in various structural and kinetic aspects. The gene family has members in various protozoan species [92,144], as well as in plants and yeast [92,137]. Sequencing of several genomes of protozoan parasites revealed the presence of genes encoding putative NTPDases [92]; [152]. However, as pointed out by Nakaar et al. ([153], see also [92]), it is sometimes difficult to clarify which gene encoding a putative NTPDase, or else another nucleotidase, is responsible for the observed nucleotide hydrolysis.

A few well-known protozoan models will be analyzed below, with particular focus on pathogenic species of genera *Toxoplasma*, *Trypanosoma*, and *Leishmania*.

In the last 25 years, evidence has been accumulating that expression of E-NTPDase genes is required for virulence of many pathogens [93,154,155,156,157]. Thus it appears likely that protozoan NTPDases, by altering the concentration of ATPe and other extracellular nucleotides accumulating on the cell surface of hosts may interfere with P signaling to suppress inflammatory responses and evade immune reactions [154].

### 4.1. *Toxoplasma gondii*

*T. gondii* encodes three sequences compatible with E-NTPDases on its genome [84], although cDNA analysis showed that only genes for TgNTPDases 1 and 2 [92], also termed NTPDases 3 and 1, respectively [84], are transcribed and translated. Amino acid sequences of both enzymes contain the canonical five ACRs, demonstrating that they are members of the E-NTPDase family. Despite 97% identity, both enzymes displayed different affinities for di- and trinucleotides, with TgNTPDase 1 exhibiting more than four-fold higher ATPase activity than TgNTPDase 2 [84,93,158]. Most virulent strains of *T. gondii* possess the gene encoding TgNTPDase 1, but avirulent strains carry only the gene encoding TgNTPDase2 [93,159]. Early studies showed that TgNTPDase 1 inhibition by antisense RNA compromised parasite replication, while pre-treatment of parasites with a monoclonal antibody inhibiting TgNTPDases 1 and 2 compromised invasion of the parasites into host cells, suggesting that NTPDase activity (one or both isoforms) is essential for parasite function [84,153,158].

Antibodies raised to recombinant NTPDases enabled localization of the enzymes within the parasite and during infection of host. Accordingly, TgNTPDases 1 and 2 are secreted into the lumen of the PV [160,161]. TgNTPDase 1 has only low levels of expression in the bradyzoite form of the parasite (the form responsible for chronic host infection) but is highly expressed in secretory granules of the actively replicating tachyzoite form [92,158].

Whether TgNTDPase activity contributes to the invasion of the parasite, and/or egress from the host, remains controversial [92,153,162]. As ATPe from the host can activate specific P2X7 receptors [12] to promote inflammation, we might assume that *T. gondii* NTPDases might likely interfere with the host inflammatory response. However, the intracellular location of TgNTPDases and their millimolar Km values [84] argue against this hypothesis. Alternatively, a role in purine salvage has been proposed. This is because TgNTPDase2 hydrolyzes ATP and ADP at similar rates. If coupled to host 5′-ecto-nucleotidase activity and other downstream ecto-enzymes, it may produce adenosine and by-products capable of being transported to the parasite (see Section 5) [163].

In the closely related *Neospora caninum*, NTPase (NcNTPDase) lacks nucleoside diphosphate hydrolase activity and appears to be more abundant in virulent isolates, suggesting a role in the pathogenicity of neosporosis, which causes abortion in cattle and neuromuscular disorders in canids [164]. Protein dynamics, secretion, and mRNA expression profiles and enzyme immunolocalization during the tachyzoite lytic cycle was compatible with NcNTPase, similarly to TgNTPDase, being localized in dense granules and the PVM throughout the lytic cycle. Up-regulation and secretion of NcNTPDase occurred during the egress and early invasion phases.

### 4.2. *Trypanosoma cruzi*

*T. cruzi* is the ethiological agent of Chagas disease, commonly spread to humans and more than 100 species of mammals by hematophagous triatomine insects. It is a cause of morbidity and mortality not only in endemic areas of North, Central, and South America but also among immigrants now residing in non-endemic areas of the world [165]. Up to date there are no vaccines or effective treatment for the disease [166]. Vector control is the most effective method of prevention. Blood screening is necessary to prevent infection through transfusion and organ transplantation [167].

Parasites circulate through the blood of the host in the form of trypomastigotes. When the triatomine insect bites and feeds on infected blood, the trypomastigote matures and differentiates to the epimastigote form, which attaches to the intestinal wall and divides. Three to four weeks later epimastigotes convert to infective trypomastigotes that move to the hindgut of the insect. Transmission to the new host occurs when parasites from insect feces enter cells at bite wound sites [165]. Parasite initially replicates within local tissues before disseminating in the bloodstream, where they can infect at distal sites [165].

*T. cruzi* encodes a single predicted NTPDase (TcNTPDase) containing all five ACRs. The fact that a protein on the parasite surface reacted with a specific antibody anti TcNTPDase suggests the surface localization of the enzyme, although a predicted N-terminal signal peptide may allow the enzyme to be secreted [168].

A recombinant form of TcNTPDase promoted hydrolysis of ATP and ADP, with an ATPase:ADPase ratio of approximately 2, i.e., similar to that of E-NTPDase 1 of mammalian models [166]. However, although E-NTPDase activity was clearly shown by exposing intact live parasites to extracellular nucleotides, problems arise when trying to assign the experimentally measured ecto-nucleotidase activities to the identity of the enzymes. For example, the ATPase:ADPase ratio was shown to vary during passage in culture, changing from 2:1 for trypomastigotes to 1:1 for epimastigotes. Moreover ARL67156, a generic ecto-nucleotidase inhibitor, partially inhibited ATP diphosphohydrolase activity, but failed to inhibit recombinant TcNTPDase [166]. On the other hand, gadolinium and suramin caused decreased infectivity in vitro and decreased virulence in a mouse model of disease [92,166], but these compounds may also interfere with ATP transport mechanisms and P receptor signaling ([157,159,169,170], see also [171]). Nevertheless, the fact that ecto-nucleotidase activities could be measured in vitro using different nucleotides (GDP, GTP, UDP, and UTP) suggests the participation of one or more NTPDases [172,173]. Moreover anti-TcNTPDase 1 polyclonal antiserum was shown to partially inhibit ATPDase activity and infectivity of trypomastigotes [166]. In addition to NTDPase activities, in vitro experiments showed that *T. brucei* has the ability to hydrolyse extracellular AMP to adenosine, indicating 5′-ecto-nucleotidase activity in this parasite [174].

More recently, Santos et al. [166] showed that high ratio of ATP/ADP extracellular hydrolysis is important to maintain the capacity of parasites to infect mammalian Vero cells lines (carcinoma-derived African green monkey fibroblast cells). Moreover, inhibition of parasite NTPDase modulated the infectivity and virulence in mice, suggesting that this enzyme might facilitate *T. cruzi* infection.

In contrast to *T. cruzi*, *T. brucei* is an extracellular pathogen that replicates in the bloodstream of mammals [175], causing African sleeping sickness. The genome of *T. brucei* encodes 2 predicted NTPDases containing N-terminal signal peptides, implying secretion by the parasite [176]. When intact parasites were exposed to exogenous nucleotides, hydrolysis of ATP, GTP, CTP, UTP, and ADP was found. In principle, the availability of efficient RNAi systems in *T. brucei* could be used to assess the importance of these enzymes in virulence [177].

### 4.3. *Leishmania*

The genome of five *Leishmania* species possesses two predicted NTPDases, containing the five ACRs [178] but no kinetic characterization of these enzymes is available. One putative E-NTPDase exhibits a predicted N-terminal transmembrane domain, compatible with anchoring of the enzyme on the membrane of the cell or that of an intracellular organelle. Another putative *Leishmania* NTPDase has a predicted N-terminal signal peptide, suggesting a potential secretion of the protein that could be responsible for extracellular nucleotide hydrolysis [179]. While it is not confirmed that the observed enzyme activity is due to members of the NTPDase family, the kinetic characterization is in principle consistent with the presence of NTPDases, although the enzymes identified in *L. tropica* and *L. amazonensis* cannot utilize Ca^2+^ instead of Mg^2+^, which is unusual for the E-NTPDase family [156,180].

In several *Leishmania* species ATP can be hydrolysed to adenosine at the cell surface, indicating the presence of NTPDase and 5′-ecto-nucleotidase activity [135]. Nucleotidase activity is higher in virulent strains than avirulent strains and is increased more than 10-fold in the obligate intracellular amastigote stage [155,156]. On the other hand, treatment of parasites with anti-CD39 antibody (i.e., cloned E-NTPDase 1) reduced the interaction of the parasites with mouse peritoneal macrophages [156], further suggesting a role for an NTPDase in pathogenesis. Moreover, the expression of ecto-nucleotidases in *Leishmania* is linked to lesion size upon infection, with high expression of these enzymes associated to decreased immune response and bigger lesions [18].

### 4.4. *Plasmodium falciparum*

Recently Borges-Pereira et al. [181] provided the first characterization of an NTPDase of *P. falciparum*. In this species, the genome predicts a gene encoding a putative E-NTPDase (Pf apyrase, PF3D7_1431800). ATPase activity as well as mRNA levels were confirmed at all stages of development, i.e., rings, trophozoites and schizonts. In isolated parasites, ATPe hydrolysis was inhibited by well-known E-NTPDase inhibitors, which strongly impaired parasite development. Expression of a N-terminal PfNTPDase-GFP chimera suggested that the NTPDase is expressed throughout the asexual cycle, but localization appears to change from the endoplasmic reticulum to the digestive vacuole as the parasite develops. Since the chimera lacks the C-terminal transmembrane sequence, the exact localization of the enzyme awaits confirmation [181]. Enzyme kinetics results suggest that PfNTPDase might translocate to the host membrane. This is because uninfected RBCs have a very low ATPe hydrolysis rate by ecto-ATPase activity, while infected RBC displayed several fold higher values at all stages [14]. Moreover, ecto-ATPase activity of trophozoite infected RBCs is approximately 400-fold higher than similar activity of uninfected cells, both at low nanomolar as well as high micromolar ATP concentrations [14]. This finding supports the hypothesis that infected cells express higher levels of functional ecto-nucleotidases than uninfected cells. Up-regulation of host E-NTPDase is unlikely, since mature RBCs lack nucleus and thus the synthesis of new proteins is restricted to the translation of pre-existing traces of mRNA. Elevated host E-NTDPase may be partly explained by a decreased degradation rate of the infected RBCs and/or posttranslational modifications. In this line, we observed before [14] that trophozoite infected RBCs can highly produce nitric oxide, which modulates host ATPe hydrolysis rate, probably by S-nitrosylation of E-NTPDase. Alternatively, PfNTPDase or an unknown parasite nucleotidase, might account to the observed elevated ecto-ATPase activity of infected cells.

**Figure 2:**
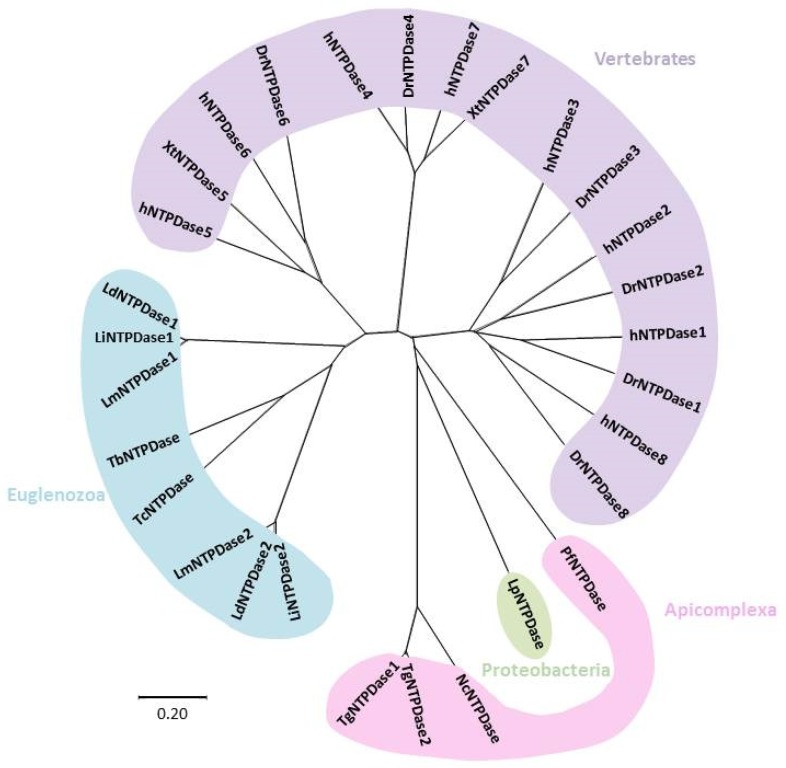
Radial phylogenetic tree from amino acid sequences of NTPDases. NTPDases from protozoan species are shown, together with NTPDases from other vertebrate species and a bacterium. The tree was generated using the ClustalW sequence alignment tool, with the resulting tree being built with the MEGA-X software, using the Neighbor-Joining method. Visualization was done with the program Figtree. The tree was drawn to scale, with branch lengths in the same units as those of the evolutionary distances used to infer the phylogenetic tree. The evolutionary distances were computed using the Poisson correction method and are in the units of the number of amino acid substitutions per site. NTPDases and their accession numbers are listed as follows. Human NTPDases: hNTPDase 1: NP_001767, hNTPDase2:NP_982293, hNTPDase3:NP_982293, hNTPDase4:NP_004892, hNTPDase5:NP_001240, hNTPDase6:NP_001238, hNTPDase7:NP_065087, hNTPDase8:NP_001028285. *Danio rerio* NTPDases: DrNTPDase 2:54261809, DrNTPDase 3: ABR15509, DrNTPDase4:50539906, DrNTPDase 6:62955697, DrNTPDase 8:268837940. *Xenopus tropicalis* NTPDases: XtNTPDase5:301618468, XtNTPDase7:62859996. Protozoan NTPDases for *Plasmodium falciparum* (Pf): PfNTPDase:AAN36910; *Toxoplasma gondii* (Tg): TgNTPDase 1: Q27893, TgNTPDase 2:Q27895; *Toxoplasma brucei* (Tc): TbNTPDase: XP_847211; *Leishmania major* (Lm): LmNTPDase1: XP_001681917, LmNTPDase2:CAJ02396; *Leishmania donovani* (Ld): LdNTPDase1:CBZ32820.1, LdNTPDase2:CBZ32136.1; *Leishmania infantum* (Li): LiNTPDase1:XP_001464341, LiNTPDase2:XP_001463665; *Trypanosoma cruzi* (Tc): TcNTPDase: AA575599; *Neospora caninum* (Nc): NcNTPDase: BAA31454. Aligments of aminoacid sequences of NTPDases are given in Supplementary Figure S1.

An evolutionary tree depicting the evolution of NTPDases is shown in Figure 2 (sequence alignment of NTPDases are given in Figure S1). Evolution of NTPDases from humans and other vertebrates shows a clear separation of subtypes locating at the cell surface (i.e., NTPDases 1–3, 8) or intracellularly (i.e., NTPDases 4–7), which correlates well with the topological and structural differences between the two groups [6]. In fact, hNTPDase1 and hNTPDase2 proved sufficiently related to form catalytically active protein chimeras [6].

Similarly to vertebrates, all protozoan ecto-nucleotidases are denoted in the tree as NTPDases, as they contain the five ACRs. As expected, species from Euglenozoa (*Trypanosoma* and *Leishmania*) showed evolutionary proximity, and the same is true for species from Apicomplexa (*Toxoplasma, Neospora and Plasmodium)*. *Toxoplasma* and *Neospora* NTPDases are consistently grouped, as TgNTPases 1 and 2 share 97% identity, while *N. caninum* exhibit 69% identity with TgNTPDase 1 [92]. Most bacteria lack NTPDases, but NTPDase from *Legionella pneumophila*, a pathogenic bacterium, was possibly acquired by horizontal transfer [6].

## 5. Nucleoside Transport

Although ATPe and other extracellular nucleotides are usually not taken up by cells, dephosphorylated nucleoside derivatives interact with specific transporters to enable intracellular uptake via specific nucleoside transporter proteins (NTs).

There are two different gene families of NTs [182]. In humans, the *SLC28* gene family encodes Na^+^ dependent concentrative nucleoside transporters, with three isoforms (CNT1–3) [182], whereas the *SLC29* gene family encodes nucleoside transporters comprising four isoforms in humans called equilibrative nucleotide transporters (ENT) 1–4. ENTs are glycosylated, possess 11 transmembrane helices with cytoplasmatic N-and extracellular C-termini, a large extracellular loop between transmembrane domains 1 and 2, and a large intracellular loop connecting transmembrane domains 6 and 7. First thought as being exclusively equilibrative transporters of nucleosides, later findings show that some family members also transport nucleobases whereas others act as proton-dependent concentrative transporters [183,184,185,186].

Recycling of nucleosides and nucleobases by NTs is essential for the synthesis of nucleotides and nucleic acids, intracellular signaling pathways (e.g., cAMP, cGMP), and phospholipid synthesis. In particular, adenosine uptake can modulate P signaling, in that it decreases the effective adenosine concentration at the cell surface, thereby terminating its action on P1 receptors [187]. Nucleoside uptake and subsequent intracellular anabolic reactions can also improve the energetic state of a cell, which sometimes translates into higher intracellular ATP concentrations. This is especially important for conductive ATP release (see Section 3), where intracellular ATP acts as the main force driving the efflux of this nucleotide [113] by protozoan and host transporters.

Following the first molecular characterizations of ENTs in human tissues (1997, in [188]), the identification of homologous proteins by functional cloning and genome analysis has revealed that the family is widely distributed in eukaryotes [188]. In several protozoa, nucleoside and nucleobase transporters are ENT family members displaying the canonical eleven transmembrane domains [189].

Structure–function relationships of parasitic protists have been reviewed recently [189]. Two NTs, LdNT1 and 2, were found in cell membrane of *Leishmania donovani*, transporting adenosine and pyrimidine nucleosides (LdNT1) and inosine and guanosine nucleosides (LdNT2) [189]. Interestingly, these transports exhibited a 100-fold higher affinity for their substrates than mammalian NTs, thus enabling an efficient use of intracellular nucleosides from the host. Expression in *Xenopus* oocytes showed LdNTs to use a proton gradient for secondary active transport of nucleosides, i.e., a concentrative mechanism qualitatively different from passive equilibrative transport of mammalian ENTs. Mutational analysis showed that a single amino acid substitution can affect substrate specificity, as substitution G183D in a transmembrane domain decreased adenosine transport, while G183A impair pyrimidine transport [189].

In *Trypanosoma brucei*, ENT transporters, include TbNT1 transporting adenine and adenosine and TbNT2-10 transporting purine nucleosides and nucleobases [183,190,191].

A low affinity adenosine ENT transporter was found in *T. gondii*, which may couple well with ecto-nucleotidases, since TgNTPDase 1 (see Section 4) promotes ATP and ADP hydrolysis at similar rates, producing AMP, which may then be a substrate of ecto 5′-nucleotidases producing adenosine, which can then be scavenged by ENT for growth [84].

### Purine Salvage of Parasitic Protozoa

Most genetic and molecular studies on ENTs have been done in parasitic protozoa, which usually lack the early ATP-dependent steps in the de novo biosynthesis of purines [192] and hence import the missing substrates using ENTs. The need to grow rapidly imposes an energetic burden to these organisms, which require ATP as a source of energy and large amounts of purines for DNA and RNA synthesis. Several reports showed that functional ENTs transporters allow parasites to salvage a wide range of nucleoside and nucleobases from the host, later used to synthetize nucleotides within the parasite [183,193]. Proteins participating in the transport and metabolism of purine nucleosides differ considerably between the human host cells and protozoa, making this pathway a potential target for drug development [8,194,195]. In contrast, parasitic protozoa are capable of synthesizing pyrimidines, with the exception of *Giardia lamblia*, *Tritrichomonas foetus*, and *Trichomonas vaginalis* [196].

In mature human RBCs infected with *Plasmodia*, nucleoside transport and metabolism is more complex, as these host cells lack purine and pyrimidine biosynthetic pathways [197], and therefore largely depend on nucleoside uptake by human ENTs (hENTs). During the intraerythrocytic life cycle of the parasite, purine transport activity of hENT is complemented by the activation of NPPs, thus ensuring the entrance of nucleosides—and other nutrients—in the cytosol. Hypoxanthine, inosine and adenosine are transported across the PV through unspecific pores [8,198], and then taken up through NTs located at the plasma membrane of the parasite. Although four putative transporters (PfNT1-4) were identified in *P. falciparum* genome, isoform 1 is mainly used for purine salvage [192]. Accordingly, protein mass spectrometry shows that PfENT1 is expressed at all parasite stages, while PfENT1 knockout parasites are not viable in RBC cultures containing micromolar concentrations of purines similar to those found in plasma [192]. Moreover, genetic disruption analysis confirmed that the *PfNT1* gene is essential for purine nucleoside uptake and parasite survival. Once inside the parasite, a series of enzymes such as *N*-ribosyltransferases and phosphotransferases will metabolize host purines to supply parasite needs.

## 6. Summary and Conclusions

ATPe dynamics of protozoan parasites was studied in terms of the proteins involved in purinergic signaling, transport and metabolism of ATP and purine salvage, with special focus on the role of protozoan proteins. Existing data show that the relative contribution of parasites versus host proteins in mediating these processes is still controversial.

Although extracellular nucleotides are known to mediate a wide range of metabolic and physiological processes in these organisms, only a few protozoan P2X-like receptors were described, with the most studied case being the DdP2X receptor of *D. discoideum*. A gene was identified encoding a P2X-like protein. Site directed mutagenesis studies, together with assessment of whole cell currents showed conservation of structure and function of a typical P2X channel. DdP2X appears to localize exclusively in an intracellular vacuole, with the ATP binding site in the lumen [57]. It remains to be seen if this intracellular localization of the receptor, possibly sensing and reacting to intravacuolar ATP, is also observed in other protozoa. Adaptation of the parasite host sometimes required up-regulation and/or activation of P2 receptors, especially P2X7 receptor involved in antimicrobial responses. In addition, polymorphisms of P2X7 receptor were identified in humans that influence the outcome of infection to toxoplasmosis, no matter the parasite genotype [90].

Only in the case of protozoan ecto-nucleotidases there is ample molecular, kinetic, and cellular information from various protozoan species, both studied in isolated conditions or interacting with their host. In most studied species, predicted NTPDases encoded in the genomes were found. Enzymes were shown to either remain attached to the parasite membrane as ecto-enzymes, or be released to their extracellular milieu. Parasite NTPDases contributed differently to ATPe regulation depending on substrate availability, the kinetic characteristics of the enzyme(s) and the localization and interaction with the host. In general, NTPDase activity seems to correlate with the virulence strength of the parasite strain. A role for these enzymes is clearly shown in *Leishmania*, where antisense RNA and antibodies raised against parasite NTPDases impaired replication and compromised host invasion [84,153,158]. Whether parasite NTPDase activity interferes with the host inflammatory response and plays a role in purine salvage should be studied in more detail.

ATPe and by products not only signal through purinergic signaling, but following ecto-nucleotidase activity they can be degraded sequentially to nucleosides and purines. In the metabolic competition of parasites and their hosts, parasites developed high performance purine salvage mechanisms, which are especially important for apicomplexan purine auxotrophs [192]. Two main strategies evolved in protozoa. First, as shown in *L. donovani* NTs, nucleoside uptake is driven actively, by means of a proton gradient [183,185]. This is unlike mammalian NTs taking purines by facilitated diffusion, which makes their activity dependent on building a favorable transmembrane gradient. Second, parasite NTs may have a several fold higher affinity for their substrates than mammalian counterparts [189].

An illustrative example of the multiple interactions of ATPe dynamics of *P. falciparum* infected human RBCs is shown in Figure 1, where both parasite and the host are purine auxotrophs.

For these and yet to discover protozoan proteins, bioinformatic tools, the design of specific ligands as well as co-immunoprecipitation studies are required to identify putative interacting proteins forming functional enzymes, receptors and transporters. Moreover, trafficking of parasite proteins to the host cell membrane deserves further investigation [68]. From a medical perspective, high throughput screening of potent and specific inhibitors against targeted parasite proteins might aid in finding drugs that kill parasites effectively, and have appropriate absorption, distribution, metabolism, and safety in humans [68].

## Supplementary Materials

The following are available online at http://www.mdpi.com/2073-4425/10/1/16/s1, Figure S1: Sequence alignment of NTPDases used to build the evolutionary tree of Figure 2.

## References

[1] Ramdani G., Langsley G. (2014). ATP, an extracellular signaling molecule in red blood cells: A messenger for malaria?. Biomed. J..

[2] Burnstock G., Verkhratsky A. (2012). Purinergic Signalling and the Nervous System.

[3] Burnstock G. (2014). Purinergic signalling: From discovery to current developments. Exp. Physiol..

[4] Li A., Banerjee J., Leung C.T., Peterson-Yantorno K., Stamer W.D., Civan M.M. (2011). Mechanisms of ATP release, the enabling step in purinergic dynamics. Cell. Physiol. Biochem..

[5] Taruno A. (2018). ATP release channels. Int. J. Mol. Sci..

[6] Zimmermann H., Zebisch M., Sträter N. (2012). Cellular function and molecular structure of ecto-nucleotidases. Purinergic Signal..

[7] Yegutkin G.G. (2014). Enzymes involved in metabolism of extracellular nucleotides and nucleosides: Functional implications and measurement of activities. Crit. Rev. Biochem. Mol. Biol..

[8] De Koning H.P., Bridges D.J., Burchmore R.J.S. (2005). Purine and pyrimidine transport in pathogenic protozoa: From biology to therapy. FEMS Microbiol. Rev..

[9] Coutinho-Silva R., Corrêa G., Sater A.A., Ojcius D.M. (2009). The P2X7 receptor and intracellular pathogens: A continuing struggle. Purinergic Signal..

[10] Coutinho-Silva R., Ojcius D.M. (2012). Role of extracellular nucleotides in the immune response against intracellular bacteria and protozoan parasites. Microbes Infect..

[11] Lebrun M., Blanchard N. (2017). Editorial overview: Host–microbe interactions: Parasites. Curr. Opin. Microbiol..

[12] Savio L.E.B., de Mello P.A., da Silva C.G., Coutinho-Silva R. (2018). The P2X7 receptor in inflammatory diseases: Angel or demon?. Front. Pharmacol..

[13] Akkaya C., Shumilina E., Bobballa D., Brand V.B., Mahmud H., Lang F., Huber S.M. (2009). The *Plasmodium falciparum*-induced anion channel of human erythrocytes is an ATP-release pathway. Pflugers Arch. Eur. J. Physiol..

[14] Alvarez C.L., Schachter J., de Sá Pinheiro A.A., de Souza Silva L., Verstraeten S.V., Persechini P.M., Schwarzbaum P.J. (2014). Regulation of extracellular ATP in human erythrocytes infected with *Plasmodium falciparum*. PLoS ONE.

[15] Levano-Garcia J., Dluzewski A.R., Markus R.P., Garcia C.R.S. (2010). Purinergic signalling is involved in the malaria parasite *Plasmodium falciparum* invasion to red blood cells. Purinergic Signal..

[16] Tanneur V., Duranton C., Brand V.B., Sandu C.D., Akkaya C., Kasinathan R.S., Gachet C., Sluyter R., Barden J.A., Wiley J.S. (2006). Purinoceptors are involved in the induction of an osmolyte permeability in malaria-infected and oxidized human erythrocytes. FASEB J..

[17] Fleck S.L., Birdsall B., Babon J., Dluzewski A.R., Martin S.R., Morgan W.D., Angov E., Kettleborough C.A., Feeney J., Blackman M.J. (2003). Suramin and suramin analogues inhibit merozoite surface protein-1 secondary processing and erythrocyte invasion by the malaria parasite *Plasmodium falciparum*. J. Biol. Chem..

[18] de Souza M.C., De Assis E.A., Saar Gomes R., De Almeida Marques-Da-Silva E., Melo M.N., Lopes Rangel Fietto J., Crocco Alfonso L.C., Afonso C. (2010). The influence of ecto-nucleotidases on *Leishmania amazonensis* infection and immune response in C57B/6 mice. Acta Trop..

[19] Leite P.M., Gomes R.S., Figueiredo A.B., Serafim T.D., Tafuri W.L., de Souza C.C., Moura S.A.L., Fietto J.L.R., Melo M.N., Ribeiro-Dias F. (2012). Ecto-nucleotidase activities of promastigotes from *Leishmania (Viannia*) *braziliensis* relates to parasite infectivity and disease clinical outcome. PLoS Negl. Trop. Dis..

[20] Khakh B.S., Alan North R. (2006). P2X receptors as cell-surface ATP sensors in health and disease. Nature.

[21] Burnstock G. (2015). Blood cells: An historical account of the roles of purinergic signalling. Purinergic Signal..

[22] Coddou C., Yan Z., Obsil T., Huidobro-Toro J.P., Stojilkovic S.S. (2011). Activation and regulation of purinergic P2X receptor channels. Pharmacol. Rev..

[23] Burnstock G., Ralevic V., Burnstock G. (1998). Receptor for purines and pyrimidines. Pharmacol. Rev..

[24] North R.A. (2002). Molecular physiology of P2X receptors. Physiol. Rev..

[25] Espelt M.V., de Tezanos Pinto F., Alvarez C.L., Alberti G.S., Incicco J., Leal Denis M.F., Davio C., Schwarzbaum P.J. (2013). On the role of ATP release, ectoATPase activity, and extracellular ADP in the regulatory volume decrease of Huh-7 human hepatoma cells. Am. J. Physiol. Cell Physiol..

[26] Pafundo D.E., Alvarez C.L., Krumschnabel G., Schwarzbaum P.J. (2010). A volume regulatory response can be triggered by nucleosides in human erythrocytes, a perfect osmometer no longer. J. Biol. Chem..

[27] Fountain S.J. (2013). Primitive ATP-activated P2X receptors: Discovery, function and pharmacology. Front. Cell. Neurosci..

[28] Kaczmarek-Hájek K., Lörinczi É., Hausmann R., Nicke A. (2012). Molecular and functional properties of P2X receptors-recent progress and persisting challenges. Purinergic Signal..

[29] Newbolt A., Stoop R., Virginio C., Surprenant A., North R.A., Buell G., Rassendren F. (1998). Membrane topology of an ATP-gated ion channel (P2X receptor). J. Biol. Chem..

[30] Abbracchio M.P., Burnstock G., Verkhratsky A., Zimmermann H. (2009). Purinergic signalling in the nervous system: An overview. Trends Neurosci..

[31] Wang J., Yu Y. (2016). Insights into the channel gating of P2X receptors from structures, dynamics and small molecules. Acta Pharmacol. Sin..

[32] Nicke A., Bäumert H.G., Rettinger J., Eichele A., Lambrecht G., Mutschler E., Schmalzing G. (1998). P2X1 and P2X3 receptors form stable trimers: A novel structural motif of ligand-gated ion channels. EMBO J..

[33] Chaumont S., Jiang L.H., Penna A., North R.A., Rassendren F. (2004). Identification of a trafficking motif involved in the stabilization and polarization of P2X receptors. J. Biol. Chem..

[34] Volonté C., D’Ambrosi N. (2009). Membrane compartments and purinergic signalling: The purinome, a complex interplay among ligands, degrading enzymes, receptors and transporters. FEBS J..

[35] Ding S., Sachs F. (1999). Single channel properties of P2X _2_ purinoceptors. J. Gen. Physiol..

[36] Jiang L.-H., Spelta V., Bo X., Surprenant A., North R.A. (2003). Status survey of the forest leopard (*Panthera pardus* Linnaeus, 1758) in Nepal. Subunit Arrangement P2X Receptors..

[37] Alvarez C.L., Corradi G., Lauri N., Marginedas-Freixa I., Leal Denis M.F., Enrique N., Mate S.M., Milesi V., Ostuni M.A., Herlax V. (2017). Dynamic regulation of extracellular ATP in *Escherichia coli*. Biochem. J..

[38] Verkhratsky A., Burnstock G. (2014). Biology of purinergic signalling: Its ancient evolutionary roots, its omnipresence and its multiple functional significance. BioEssays.

[39] Fountain S.J., Cao L., Young M.T., North R.A. (2008). Permeation properties of a P2X receptor in the green algae *Ostreococcus tauri*. J. Biol. Chem..

[40] Hefetz A., Keeling C.I., Winston M.L., Slessor K.N., Nielsen J., Lanfear R., Liebig J., Millar J.G., Hanks L.M., Suarez A.V. (2014). Identification of a plant receptor for extracellular ATP. Science.

[41] Clark G., Roux S.J. (2011). Apyrases, extracellular ATP and the regulation of growth. Curr. Opin. Plant Biol..

[42] Ludlow M.J., Ludlow M.J. (2008). Purinergic signalling in *Dictyostelium discoideum*. Ph.D. Thesis.

[43] Fountain S.J., Parkinson K., Young M.T., Cao L., Thompson C.R.L., North R.A. (2007). An intracellular P2X receptor required for osmoregulation in *Dictyostelium discoideum*. Nature.

[44] Nakazawa K., Ohno Y. (1999). Neighboring glycine residues are essential for P2X2 receptor/channel function. Eur. J. Pharmacol..

[45] Agboh K.C., Webb T.E., Evans R.J., Ennion S.J. (2004). Functional characterization of a P2X receptor from *Schistosoma mansoni*. J. Biol. Chem..

[46] Hayasi M., Takahashi M. (1979). Ciliary adenosinetriphosphatase from a slow swimming mutant of *Paramecium caudatum*. J. Biol. Chem. Biol. Chem..

[47] Kim M.Y., Kuruvilla H.G., Raghu S., Hennessey T.M. (1999). ATP reception and chemosensory adaptation in *Tetrahymena thermophila*. J. Exp. Biol..

[48] Inverso J.A., Song Y., Santos-Buch C.A. (1995). Plasma membrane ATP receptors in *Trypanosoma cruzi* trypomastigotes. Receptor.

[49] Pothier F., Forget J., Sullivan R., Couillard P. (1987). ATP and the contractile vacuole in *Amoeba proteus*: Mechanism of action of exogenous ATP and related nucleotides. J. Exp. Zool..

[50] Uyeda T.Q.P., Furuya M. (1987). ATP-induced relative movement between microfilaments and microtubules in myxomycete flagellates. Protoplasma.

[51] Yue J., Sun G., Hu X., Huang J. (2013). The scale and evolutionary significance of horizontal gene transfer in the choanoflagellate *Monosiga brevicollis*. BMC Genom..

[52] King N., Westbrook M.J., Young S.L., Kuo A., Abedin M., Chapman J., Fairclough S., Hellsten U., Isogai Y., Letunic I. (2008). The genome of the choanoflagellate *Monosiga brevicollis* and the origin of metazoans. Nature.

[53] Eichinger I., Pachebat J.A., Glöckner G., Rajandream M.A., Sucgang R., Berriman M., Song J., Olsen R., Szafranski K., Xu Q. (2005). The genome of the social amoeba *Dictyostelium discoideum*. Nature.

[54] Leal Denis M.F., Incicco J.J., Espelt M.V., Verstraeten S.V., Pignataro O.P., Lazarowski E.R., Schwarzbaum P.J. (2013). Kinetics of extracellular ATP in mastoparan 7-activated human erythrocytes. Biochim. Biophys. Acta Gen. Subj..

[55] Leal Denis M.F., Alvarez H.A., Lauri N., Alvarez C.L., Chara O., Schwarzbaum P.J. (2016). Dynamic regulation of cell volume and extracellular ATP of human erythrocytes. PLoS ONE.

[56] Surprenant A., North R.A. (2009). Signaling at Purinergic P2X Receptors. Annu. Rev. Physiol..

[57] Sivaramakrishnan V., Fountain S.J. (2015). Evidence for extracellular ATP as a stress signal in a single-celled organism. Eukaryot. Cell.

[58] Ghazanfari N., Mueller S.N., Heath W.R. (2018). Cerebral malaria in mouse and man. Front. Immunol..

[59] Cowman A.F., Tonkin C.J., Tham W.H., Duraisingh M.T. (2017). The molecular basis of erythrocyte invasion by malaria parasites. Cell Host Microbe.

[60] Ashley E.A., Phyo P., Woodrow C.J. (2018). Seminar Malaria. Lancet.

[61] Gardner M.J., Hall N., Fung E., White O., Berriman M., Hyman R.W., Carlton J.M., Pain A., Nelson K.E., Bowman S. (2002). Genome sequence of the human malaria parasite *Plasmodium falciparum*. Nature.

[62] Frech C., Chen N. (2011). Genome comparison of human and non-human malaria parasites reveals species subset-specific genes potentially linked to human disease. PLoS Comput. Biol..

[63] Budu A., Garcia C.R.S. (2012). Generation of second messengers in *Plasmodium*. Microbes Infect..

[64] Maier A.G., Cooke B.M., Cowman A.F., Tilley L. (2009). Malaria parasite proteins that remodel the host erythrocyte. Nat. Rev. Microbiol..

[65] Tilley L., Sougrat R., Lithgow T., Hanssen E. (2008). The twists and turns of Maurer’s cleft trafficking in *P. falciparum*-infected erythrocytes. Traffic.

[66] Desai S.A. (2014). Why do malaria parasites increase host erythrocyte permeability?. Trends Parasitol..

[67] Gupta A., Balabaskaran-nina P., Nguitragool W., Saggu G.S., Schureck M.A., Desai A. (2018). CLAG3 Self-associates in malaria parasites and quantitatively determines nutrient uptake channels at the host membrane. Am. Soc. Microbiol..

[68] Chalapareddy S., Desai S.A. (2017). Malaria parasite proteins involved in nutrient channels at the host erythrocyte membrane: Advances and questions for future research. Int. J. Curr. Multidiscip. Stud..

[69] Marginedas-Freixa I., Hattab C., Bouyer G., Halle F., Chene A., Lefevre S.D., Cambot M., Cueff A., Schmitt M., Gamain B. (2016). TSPO ligands stimulate ZnPPIX transport and ROS accumulation leading to the inhibition of *P. falciparum* growth in human blood. Sci. Rep..

[70] Brochet M., Billker O. (2016). Calcium signalling in malaria parasites. Mol. Microbiol..

[71] Zipprer E.M., Neggers M., Kushwaha A., Rayavara K., Desai S.A. (2014). A kinetic fluorescence assay reveals unusual features of Ca^++^ uptake in *Plasmodium falciparum*-infected erythrocytes. Lipids Health Dis..

[72] Wasserman M., Alarcón C., Mendoza P.M. (1982). Effects of Ca^++^ depletion on the asexual cell cycle of *Plasmodium falciparum*. Am. J. Trop. Med. Hyg..

[73] Cruz L., Juliano M., Budu A., Juliano L., Holder A.A., Blackman M.J., Garcia C.R. (2012). Extracellular ATP triggers proteolysis and cytosolic Ca^2+^ rise in *Plasmodium berghei* and *Plasmodium yoelii* malaria parasites. Malar. J..

[74] Kanaani J., Ginsburg H. (1988). Compartment analysis of ATP in malaria-infected erythrocytes. Biochem. Int..

[75] Gorman M.W., Feigl E.O., Buffington C.W. (2007). Human plasma ATP concentration. Clin. Chem..

[76] Gazarini M.L., Thomas A.P., Pozzan T., Garcia C.R.S. (2003). Calcium signaling in a low calcium environment: How the intracellular malaria parasite solves the problem. J. Cell Biol..

[77] Adovelande J., Bastide B., Délèze J., Schrével J. (1993). Cytosolic free calcium in *Plasmodium falciparum*-infected erythrocytes and the effect of verapamil: A cytofluorimetric study. Exp. Parasitol..

[78] Garcia C.R., Dluzewski A.R., Catalani L.H., Burting R., Hoyland J., Mason W.T. (1996). Calcium homeostasis in intraerythrocytic malaria parasites. Eur. J. Cell Biol..

[79] Kushwaha A.K., Apolis L., Ito D., Desai S.A. (2018). Increased Ca^++^ uptake by erythrocytes infected with malaria parasites: Evidence for exported proteins and novel inhibitors. Cell. Microbiol..

[80] Schlesinger P.H., Krogstad D.J., Desai S.A., McCleskey E.W. (1996). A novel pathway for Ca^++^ entry into *Plasmodium falciparum*-infected blood cells. Am. J. Trop. Med. Hyg..

[81] Miller C.M., Boulter N.R., Fuller S.J., Zakrzewski A.M., Lees M.P., Saunders B.M., Wiley J.S., Smith N.C. (2011). The role of the P2X7 receptor in infectious diseases. PLoS Pathog..

[82] Sibley L.D. (2003). *Toxoplasma gondii*: Perfecting an intracellular life style. Traffic.

[83] Montoya J.G., Liesenfeld O. (2004). Toxoplasmosis. Lancet.

[84] Krug U., Zebisch M., Krauss M., Sträter N. (2012). Structural insight into activation mechanism of *Toxoplasma gondii* nucleoside triphosphate diphosphohydrolases by disulfide reduction. J. Biol. Chem..

[85] Mercier C., Dubremetz J.-F., Rauscher B., Lecordier L., Sibley L.D., Cesbron-Delauw M.-F. (2002). Biogenesis of nanotubular network in *Toxoplasma* parasitophorous vacuole induced by parasite proteins. Mol. Biol. Cell.

[86] Laliberté J., Carruthers V.B. (2008). Host cell manipulation by the human pathogen *Toxoplasma gondii*. Cell Mol Life Sci..

[87] Corrêa G., Marques da Silva C., de Abreu Moreira-Souza A.C., Vommaro R.C., Coutinho-Silva R. (2010). Activation of the P2X7 receptor triggers the elimination of *Toxoplasma gondii* tachyzoites from infected macrophages. Microbes Infect..

[88] Lees M.P., Fuller S.J., Mcleod R., Boulter N.R., Catherine M., Zakrzewski A.M., Mui E.J., Witola W.H., Coyne J.J., Hargrave C. (2010). P2X7 receptor-mediated killing of an intracellular parasite, *Toxoplasma gondii*, by human and murine macrophages1. J Immunol..

[89] Moreira-Souza A.C.A., Almeida-da-Silva C.L.C., Rangel T.P., Rocha G.D.C., Bellio M., Zamboni D.S., Vommaro R.C., Coutinho-Silva R. (2017). The P2X7 receptor mediates *Toxoplasma gondii* control in macrophages through canonical NLRP3 inflammasome activation and reactive oxygen species production. Front. Immunol..

[90] Jamieson S.E., Peixoto-Rangel A.L., Hargrave A.C., Roubaix L.A.D., Mui E.J., Boulter N.R., Miller E.N., Fuller S.J., Wiley J.S., Castellucci L. (2010). Evidence for associations between the purinergic receptor P2X_7_ (P2RX7) and toxoplasmosis. Genes Immun..

[91] Melo M.B., Jensen K.D.C., Saeij J.P.J. (2011). *Toxoplasma gondii* effectors are master regulators of the inflammatory response. Trends Parasitol..

[92] Sansom F.M. (2012). The role of the NTPDase enzyme family in parasites: What do we know, and where to from here?. Parasitology.

[93] Asai T., Miura S., Sibley L.D., Okabayashi H., Takeuchi T. (1995). Biochemical and molecular characterization of nucleoside triphosphate hydrolase isozymes from the parasitic protozoan *Toxoplasma gondii*. J. Exp. Psychol. Gen..

[94] Coutinho-Silva R., Morandini A., Savio L.B. (2014). The role of P2X7 receptor in infectious inflammatory diseases and the influence of ectonucleotidases. Biomed. J..

[95] Alam M.S., Costales M.G., Cavanaugh C., Williams K. (2015). Extracellular adenosine generation in the regulation of pro-inflammatory responses and pathogen colonization. Biomolecules.

[96] Giuliani A.L., Sarti A.C., Di Virgilio F. (2018). Extracellular nucleotides and nucleosides as signalling molecules. Immunol. Lett..

[97] Dunay I.R., Sibley L.D. (2010). Monocytes mediate mucosal immunity to *Toxoplasma gondii*. Curr. Opin. Immunol..

[98] Säve S., Mohlin C., Vumma R., Persson K. (2011). Activation of adenosine A2Areceptors inhibits neutrophil transuroepithelial migration. Infect. Immun..

[99] Mahamed D.A., Toussaint L.E., Bynoe M.S. (2015). CD73-generated adenosine is critical for immune regulation during *Toxoplasma gondii* infection. Infect. Immun..

[100] Denkers E.Y., Butcher B.A. (2005). Sabotage and exploitation in macrophages parasitized by intracellular protozoans. Trends Parasitol..

[101] Chaves S.P., Torres-Santos E.C., Marques C., Figliuolo V.R., Persechini P.M., Coutinho-Silva R., Rossi-Bergmann B. (2009). Modulation of P2X7purinergic receptor in macrophages by *Leishmania amazonensis* and its role in parasite elimination. Microbes Infect..

[102] Chaves M.M., Marques-da-Silva C., Monteiro A.P.T., Canetti C., Coutinho-Silva R. (2014). Leukotriene B4 modulates P2X7 receptor-mediated *Leishmania amazonensis* elimination in murine macrophages. J. Immunol..

[103] Jacobson K.A., Balasubramanian R., Deflorian F., Gao Z.-G. (2012). G protein-coupled adenosine (P1) and P2Y receptors: Ligand design and receptor interactions. Purinergic Signal..

[104] Marques-da-Silva C., Chaves M.M., Chaves S.P., Figliuolo V.R., Meyer-Fernandes J.R., Corte-Real S., Lameu C., Ulrich H., Ojcius D.M., Rossi-Bergmann B. (2011). Infection with *Leishmania amazonensis* upregulates purinergic receptor expression and induces host-cell susceptibility to UTP-mediated apoptosis. Cell. Microbiol..

[105] Arora K., Rai A.K. (2018). Dependence of *Leishmania* parasite on host derived ATP: An overview of extracellular nucleotide metabolism in parasite. J. Parasit. Dis..

[106] Rai A.K., Thakur C.P., Velpandian T., Sharma S.K., Ghosh B., Mitra D.K. (2011). High concentration of adenosine in human visceral leishmaniasis despite increased ADA and decreased CD73. Parasite Immunol..

[107] De Almeida Marques-da-Silva E., de Oliveira J.C., Figueiredo A.B., de Souza Lima Júnior D., Carneiro C.M., Rangel Fietto J.L., Crocco Afonso L.C. (2008). Extracellular nucleotide metabolism in *Leishmania*: Influence of adenosine in the establishment of infection. Microbes Infect..

[108] Lima M.H.F., Sacramento L.A., Quirino G.F.S., Ferreira M.D., Benevides L., Santana A.K.M., Cunha F.Q., Almeida R.P., Silva J.S., Carregaro V. (2017). *Leishmania infantum* parasites subvert the host inflammatory response through the adenosine A2_A_ receptor to promote the establishment of infection. Front. Immunol..

[109] Mahaut-smith M.P., Jones S., Evans R.J. (2011). The P2X1 receptor and platelet function. Purinergic Signal..

[110] Angchaisuksiri P. (2014). Coagulopathy in malaria. Thromb. Res..

[111] Idzko M., Ferrari D., Eltzschig H.K. (2014). Nucleotide signalling during inflammation. NIH Public Access.

[112] Manohar M., Hirsh M.I., Chen Y., Woehrle T., Karande A.A., Junger W.G. (2012). ATP release and autocrine signaling through P2X4 receptors regulate T cell activation. J. Leukoc. Biol..

[113] Lazarowski E.R. (2012). Vesicular and conductive mechanisms of nucleotide release. Purinergic Signal..

[114] Yegutkin G.G. (2008). Nucleotide- and nucleoside-converting ectoenzymes: Important modulators of purinergic signalling cascade. Biochim. Biophys. Acta Mol. Cell Res..

[115] Praetorius H.A., Leipziger J. (2009). ATP release from non-excitable cells. Purinergic Signal..

[116] Sabirov R.Z., Merzlyak P.G. (2012). Plasmalemmal VDAC controversies and maxi-anion channel puzzle. Biochim. Biophys. Acta Biomembr..

[117] Dahl G. (2015). ATP release through pannexon channels. Philos. Trans. R. Soc. Lond. B. Biol. Sci..

[118] Huber S.M. (2012). Purinoceptor signaling in malaria-infected erythrocytes. Microbes Infect..

[119] Sabirov R.Z., Okada Y. (2005). ATP release via anion channels. Purinergic Signal..

[120] Marginedas-Freixa I., Alvarez C.L., Moras M., Leal Denis M.F., Hattab C., Halle F., Bihel F., Mouro-Chanteloup I., Lefevre S.D., Le Van Kim C. (2018). Human erythrocytes release ATP by a novel pathway involving VDAC oligomerization independent of pannexin-1. Sci. Rep..

[121] Capella-Gutierrez S., Marcet-Houben M., Gabaldón T. (2012). Phylogenomics supports microsporidia as the earliest diverging clade of sequenced fungi. BMC Biol..

[122] Didier E.S., Weiss L.M. (2011). Microsporidiosis: Not just in AIDS patients. Curr. Opin. Infect. Dis..

[123] Nakjang S., Williams T.A., Heinz E., Watson A.K., Foster P.G., Sendra K.M., Heaps S.E., Hirt R.P., Embley T.M. (2013). Reduction and expansion inmicrosporidian genome evolution: New insights from comparative genomics. Genome Biol. Evol..

[124] Dean P., Sendra K.M., Williams T.A., Watson A.K., Major P., Nakjang S., Kozhevnikova E., Goldberg A.V., Kunji E.R.S., Hirt R.P. (2018). Transporter gene acquisition and innovation in the evolution of Microsporidia intracellular parasites. Nat. Commun..

[125] Heinz E., Hacker C., Dean P., Mifsud J., Goldberg A.V., Williams T.A., Nakjang S., Gregory A., Hirt R.P., Lucocq J.M. (2014). Plasma membrane-located purine nucleotide transport proteins are key components for host exploitation by microsporidian intracellular parasites. PLoS Pathog..

[126] Schmitz-Esser S., Linka N., Collingro A., Beier C.L., Neuhaus H.E., Wagner M., Horn M. (2004). ATP/ADP translocases: A common feature of obligate intracellular amoebal symbionts related to Chlamydiae and Rickettsiae. J. Bacteriol..

[127] Bouyer G., Cueff A., Egée S., Kmiecik J., Maksimova Y., Glogowska E., Gallagher P.G., Thomas S.L.Y. (2011). Erythrocyte peripheral type benzodiazepine receptor/voltage-dependent anion channels are upregulated by *Plasmodium falciparum*. Blood.

[128] Marginedas-Freixa I., Alvarez C.L., Moras M., Hattab C., Bouyer G., Chene A., Lefevre S.D., Le Van Kim C., Bihel F., Schwarzbaum P.J. (2018). Induction of ATP Release, PPIX Transport, and cholesterol uptake by human red blood cells using a new family of TSPO ligands. Int. J. Mol. Sci..

[129] Lutz P.L., Kabler S. (1997). Release of adenosine and ATP in the brain of the freshwater turtle (*Trachemys scripta*) during long-term anoxia. Brain Res..

[130] Clarke T.C., Williams O.J.S., Martin P.E.M., Evans W.H. (2009). ATP release by cardiac myocytes in a simulated ischaemia model. Inhibition by a connexin mimetic and enhancement by an antiarrhythmic peptide. Eur. J. Pharmacol..

[131] Lim To W.K., Kumar P., Marshall J.M. (2015). Hypoxia is an effective stimulus for vesicular release of ATP from human umbilical vein endothelial cells. Placenta.

[132] Rajani V., Zhang Y., Jalubula V., Rancic V., SheikhBahaei S., Zwicker J.D., Pagliardini S., Dickson C.T., Ballanyi K., Kasparov S. (2018). Release of ATP by pre-Bötzinger complex astrocytes contributes to the hypoxic ventilatory response via a Ca^2+^-dependent P2Y_1_ receptor mechanism. J. Physiol..

[133] Sridharan M., Adderley S.P., Bowles E.A., Egan T.M., Stephenson A.H., Ellsworth M.L., Sprague R.S. (2010). Pannexin 1 is the conduit for low oxygen tension-induced ATP release from human erythrocytes. AJP Hear. Circ. Physiol..

[134] Maguire M.H., Lukas M.C., Rettie J.F. (1972). Adenine Nucleotide salvage synthesis pathways of adenosine salvage in the rat heart. Biochim. Biophys. Acta.

[135] Paes-Vieira L., Gomes-Vieira A.L., Meyer-Fernandes J.R. (2018). NTPDase activities: Possible roles on *Leishmania* spp. infectivity and virulence. Cell Biol. Int..

[136] Das S., Saha A.K., Mukhopadhyay N.K., Glew R.H. (1986). A cyclic nucleotide-independent protein kinase in *Leishmania donovani*. Biochem. J..

[137] Robson S.C., Sévigny J., Zimmermann H. (2006). The E-NTPDase family of ectonucleotidases: Structure function relationships and pathophysiological significance. Purinergic Signal..

[138] Franco R., Pacheco R., Gatell J.M., Gallart T., Lluis C. (2007). Enzymatic and extraenzymatic role of adenosine deaminase 1 in T-cell-dendritic cell contacts and in alterations of the immune function. Crit. Rev. Immunol..

[139] Yegutkin G.G., Samburski S.S., Jalkanen S. (2003). Soluble purine-converting enzymes circulate in human blood and regulate extracellular ATP level via counteracting pyrophosphatase and phosphotransfer reactions. FASEB J..

[140] Boison D. (2013). Adenosine kinase: Exploitation for therapeutic gain. Pharmacol. Rev..

[141] Virtanen S.S., Kukkonen-Macchi A., Vainio M., Elima K., Harkonen P.L., Jalkanen S., Yegutkin G.G. (2014). Adenosine Inhibits tumor cell invasion via receptor-independent mechanisms. Mol. Cancer Res..

[142] Plesner L. (1995). Ecto-ATPases: Identities and functions. Int. Rev. Cytol..

[143] Kukulski F., Levesque S.A., Lavoie E.G., Lecka J., Bigonnesse F., Knowles A.F., Robson S.C., Kirley T.L., Sevigny J. (2005). Erratum: Comparative hrydrolysis of P2 receptor agonists by NTPDases 1, 2, 3 and 8. (Purinergic Signalling (2005) vol. 1 (2) (193-204)). Purinergic Signal..

[144] Handa M., Guidotti G. (1996). Purification and cloning of a soluble ATP-diphosphohydrolase (Apyrase) from potato tubers (*Solanum tuberosum*). Biochem. Biophys. Res. Commun..

[145] Schulte Am Esch J., Sévigny J., Kaczmarek E., Siegel J.B., Imai M., Koziak K., Beaudoin A.R., Robson S.C. (1999). Structural elements and limited proteolysis of CD39 influence ATP diphosphohydrolase activity. Biochemistry.

[146] Wu J.J., Choi L.E., Guidotti G. (2005). *N*-linked oligosaccharides affect the enzymatic activity of CD39: Diverse interactions between seven *N*-linked glycosylation sites. Mol. Biol. Cell.

[147] Knowles A.F. (2011). The GDA1_CD39 superfamily: NTPDases with diverse functions. Purinergic Signal..

[148] Kirley T.L., Crawford P.A., Smith T.M. (2006). The structure of the nucleoside triphosphate diphosphohydrolases (NTPDases) as revealed by mutagenic and computational modeling analyses. Purinergic Signal..

[149] Mandapathil M., Hilldorfer B., Szczepanski M.J., Czystowska M., Szajnik M., Ren J., Lang S., Jackson E.K., Gorelik E., Whiteside T.L. (2010). Generation and accumulation of immunosuppressive adenosine by human CD4^+^CD25^high^FOXP3^+^ regulatory T cells. J. Biol. Chem..

[150] Heine P., Braun N., Sévigny J., Robson S.C., Servos J., Zimmermann H. (2001). The C-terminal cysteine-rich region dictates specific catalytic properties in chimeras of the ectonucleotidases NTPDase1 and NTPDase2. Eur. J. Biochem..

[151] Zimmermann H. (2001). Ectonucleotidases: Some recent developments and a note on nomenclature. Drug Dev. Res..

[152] EMBL SMART-Simple Modular Architecture Research Tool.

[153] Nakaar V., Samuel B.U., Ngo E.O., Joiner K.A. (1999). Targeted reduction of nucleoside triphosphate hydrolase by antisense RNA inhibits *Toxoplasma gondii* proliferation. J. Biol. Chem..

[154] Sansom F.M., Robson S.C., Hartland E.L. (2008). Possible effects of microbial ecto-nucleoside triphosphate diphosphohydrolases on host-pathogen interactions. Microbiol. Mol. Biol. Rev..

[155] Berrêdo-Pinho M., Peres-Sampaio C.E., Chrispim P.P.M., Belmont-Firpo R., Lemos A.P., Martiny A., Vannier-Santos M.A., Meyer-Fernandes J.R. (2001). A Mg-dependent ecto-ATPase in *Leishmania amazonensis* and its possible role in adenosine acquisition and virulence. Arch. Biochem. Biophys..

[156] Pinheiro C.M., Martins-Duarte E.S., Ferraro R.B., Fonseca de Souza A.L., Gomes M.T., Lopes A.H.C.S., Vannier-Santos M.A., Santos A.L.S., Meyer-Fernandes J.R. (2006). *Leishmania amazonensis*: Biological and biochemical characterization of ecto-nucleoside triphosphate diphosphohydrolase activities. Exp. Parasitol..

[157] Sansom F.M., Newton H.J., Crikis S., Cianciotto N.P., Cowan P.J., d’Apice A.J.F., Hartland E.L. (2007). A bacterial ecto-triphosphate diphosphohydrolase similar to human CD39 is essential for intracellular multiplication of *Legionella pneumophila*. Cell. Microbiol..

[158] Nakaar V., Beckers C.J.M., Polotsky V., Joiner K.A. (1998). Basis for substrate specificity of the *Toxoplasma gondii* nucleoside triphosphate hydrolase. Mol. Biochem. Parasitol..

[159] Silverman J.A., Qi H., Riehl A., Beckers C., Nakaar V., Joiner K.A. (1998). Induced activation of the *Toxoplasma gondii* nucleoside triphosphate hydrolase leads to depletion of host cell ATP levels and rapid exit of intracellular parasites from infected cells. J. Biol. Chem..

[160] Bermudes D., Peck K.R., Afifi M.A., Beckers C.J.M., Joiner K.A. (1994). Tandemly repeated genes encode nucleoside triphosphate hydrolase isoforms secreted into the parasitophorous vacuole of *Toxoplasma gondii*. J. Biol. Chem..

[161] Sibley L.D., Niesman I.R., Asai T., Takeuchi T. (1994). *Toxoplasma gondii*: Secretion of a potent nucleoside triphosphate hydrolase into the parasitophorous vacuole. Exp. Parasitol..

[162] Kikuchi T., Furuta T., Kojima S. (2001). Membrane localization and demonstration of isoforms of nucleoside triphosphate hydrolase from *Toxoplasma gondii*. Parasitology.

[163] Montalbetti N., Leal Denis M.F., Pignataros O.P., Kobatake E., Lazarowski E.R., Schwarzbaum P.J. (2011). Homeostasis of extracellular ATP in human erythrocytes. J. Biol. Chem..

[164] Pastor-Fernández I., Regidor-Cerrillo J., Álvarez-García G., Marugán-Hernández V., García-Lunar P., Hemphill A., Ortega-Mora L.M. (2016). The tandemly repeated NTPase (NTPDase) from *Neospora caninum* is a canonical dense granule protein whose RNA expression, protein secretion and phosphorylation coincides with the tachyzoite egress. Parasites Vectors.

[165] Nagajyothi F., Machado F.S., Burleigh B.A., Jelicks L.A., Scherer E., Mukherjee S., Lisanti M.P., Weiss L.M., Garg N.J., Tanowitz H.B. (2013). Mechanisms of *Trypanosoma cruzi* persistence in Chagas disease. Cell Microbiol..

[166] Santos R.F., Pôssa M.A.S., Bastos M.S., Guedes P.M.M., Almeida M.R., DeMarco R., Verjovski-Almeida S., Bahia M.T., Fietto J.L.R. (2009). Influence of ecto-nucleoside triphosphate diphosphohydrolase activity on *Trypanosoma cruzi* infectivity and virulence. PLoS Negl. Trop. Dis..

[167] WHO Chagas Disease American Trypanosomiasis. https://www.who.int/chagas/en/.

[168] Fietto J.L.R., DeMarco R., Nascimento I.P., Castro I.M., Carvalho T.M.U., De Souza W., Bahia M.T., Alves M.J.M., Verjovski-Almeida S. (2004). Characterization and immunolocalization of an NTP diphosphohydrolase of *Trypanosoma cruzi*. Biochem. Biophys. Res. Commun..

[169] Schnurr M., Then F., Galambos P., Scholz C., Siegmund B., Endres S., Eigler A. (2000). Extracellular ATP and TNF- synergize in the activation and maturation of human dendritic cells. J. Immunol..

[170] Zimmermann H. (2000). Extracellular metabolism of ATP and other nucleotides. Naunyn. Schmiedebergs. Arch. Pharmacol..

[171] Santos E.C., Novaes R.D., Cupertino M.C., Bastos D.S.S., Klein R.C., Silva E.A.M., Fietto J.L.R., Talvani A., Bahia M.T., Oliveira L.L. (2015). Concomitant benznidazole and suramin chemotherapy in mice infected with a virulent strain of *Trypanosoma cruzi*. Antimicrob. Agents Chemother..

[172] Bisaggio D.F.R., Peres-Sampaio C.E., Meyer-Fernandes J.R., Souto-Padrón T. (2003). Ecto-ATPase activity on the surface of *Trypanosoma cruzi* and its possible role in the parasite-host cell interaction. Parasitol. Res..

[173] Mariotini-Moura C., Bastos M.S. e., de Castro F.F., Trindade M.L., de Souza Vasconcellos R., Neves-do-Valle M.A.A., Moreira B.P., de Freitas Santos R., de Oliveira C.M., Cunha L.C.S. (2014). *Trypanosoma cruzi* nucleoside triphosphate diphosphohydrolase 1 (TcNTPDase-1) biochemical characterization, immunolocalization and possible role in host cell adhesion. Acta Trop..

[174] de Souza Leite M., Thomaz R., Fonseca F.V., Panizzutti R., Vercesi A.E., Meyer-Fernandes J.R. (2007). *Trypanosoma brucei brucei*: Biochemical characterization of ecto-nucleoside triphosphate diphosphohydrolase activities. Exp. Parasitol..

[175] Matthews K.R. (2005). The developmental cell biology of *Trypanosoma brucei*. J. Cell Sci..

[176] Berriman M., Ghedin E., Hertz-Fowler C., Blandin G., Renauld H., Bartholomeu D.C., Lennard N.J., Caler E., Hamlin N.E., Haas B. (2005). The genome of the African trypanosome *Trypanosoma brucei*. Science.

[177] Balaña-Fouce R., Reguera R.M. (2007). RNA interference in *Trypanosoma brucei*: A high-throughput engine for functional genomics in trypanosomatids?. TRENDS Parasitol..

[178] Peacock C.S., Seeger K., Harris D., Murphy L., Ruiz J.C., Quail M.A., Peters N., Adlem E., Tivey A., Aslett M. (2008). 2008 Comparative genomic analysis of three *Leishmania* species that cause diverse human disease. Nat. Genet..

[179] Sansom F.M., Ralton J.E., Sernee M.F., Cohen A.M., Hooker D.J., Hartland E.L., Naderer T., McConville M.J. (2014). Golgi-located NTPDase1 of *Leishmania major* is required for lipophosphoglycan elongation and normal lesion development whereas secreted NTPDase2 is dispensable for virulence. PLoS Negl. Trop. Dis..

[180] Meyer-Fernandes J.R., Dutra P.M.L., Rodrigues C.O., Saad-Nehme J., Lopes A.H.C.S. (1997). Mg-dependent ecto-ATPase activity in *Leishmania tropica*. Arch. Biochem. Biophys..

[181] Borges-Pereira L., Meissner K.A., Wrenger C., Garcia C.R.S. (2017). *Plasmodium falciparum* GFP-E-NTPDase expression at the intraerythrocytic stages and its inhibition blocks the development of the human malaria parasite. Purinergic Signal..

[182] Dos Santos-Rodrigues A., Grañé-Boladeras N., Bicket A., Coe I.R. (2014). Nucleoside transporters in the purinome. Neurochem. Int..

[183] Landfear S.M., Ullman B., Carter N.S., Sanchez M.A. (2004). Nucleoside and nucleobase transporters in parasitic protozoa. Eukaryot. Cell.

[184] Yao S.Y.M., Ng A.M.L., Cass C.E., Baldwin S.A., Young J.D. (2011). Nucleobase transport by human equilibrative nucleoside transporter 1 (hENT1). J. Biol. Chem..

[185] Landfear S.M. (2011). Nutrient transport and pathogenesis in selected parasitic protozoa. Eukaryot. Cell.

[186] Boswell-Casteel R.C., Hays F.A. (2016). Equilibrative nucleoside transporters—A review. Nucleosides Nucleotides Nucleic Acids.

[187] Löffler M., Morote-Garcia J.C., Eltzschig S.A., Coe I.R., Eltzschig H.K. (2007). Physiological roles of vascular nucleoside transporters. Arterioscler. Thromb. Vasc. Biol..

[188] Hyde R.J., Cass C.E., Young J.D., Baldwin S.A. (2001). The ENT family of eukaryote nucleoside and nucleobase transporters: Recent advances in the investigation of structure/function relationships and the identification of novel isoforms. Mol. Membr. Biol..

[189] Dean P., Major P., Nakjang S., Hirt R.P., Embley T.M. (2014). Transport proteins of parasitic protists and their role in nutrient salvage. Front. Plant Sci..

[190] Sanchez M.A., Tryon R., Green J., Boor I., Landfear S.M. (2002). Six related nucleoside/nucleobase transporters from *Trypanosoma brucei* exhibit distinct biochemical functions. J. Biol. Chem..

[191] Mäser P., Sütterlin C., Kralli A., Kaminsky R. (1999). A nucleoside transporter from *Trypanosoma brucei* involved in drug resistance. Science.

[192] Carter N.S., Mamoun C.B., Liu W., Silva E.O., Landfear S.M., Goldberg D.E., Ullman B. (2000). Isolation and functional characterization of the PfNT1 nucleoside transporter gene from *Plasmodium falciparum*. J. Biol. Chem..

[193] Carter N.S., Landfear S.M., Ullman B. (2001). Nucleoside transporters of parasitic protozoa. Trends Parasitol..

[194] Berg M., Van der Veken P., Goeminne A., Haemers A., Augustyns K. (2010). Inhibitors of the purine salvage pathway: A valuable approach for antiprotozoal chemotherapy?. Curr. Med. Chem..

[195] Cassera M.B., Zhang Y., Hazleton K.Z., Schramm V.L. (2011). Purine and pyrimidine pathways as targets in *Plasmodium falciparum*. Curr. Top. Med. Chem..

[196] Hassan H.F., Coombs G.H. (1988). Purine and pyrimidine metabolism in parasitic protozoa. FEMS Microbiol. Lett..

[197] Riegelhaupt P.M., Cassera M.B., Fröhlich R.F.G., Hazleton K.Z., Hefter J.J., Schramm V.L., Akabas M.H. (2010). Transport of purines and purine salvage pathway inhibitors by the *Plasmodium falciparum* equilibrative nucleoside transporter PfENT1. Mol. Biochem. Parasitol..

[198] Schwab J.C., Beckers C.J.M., Joiner K.A. (1994). The parasitophorous vacuole membrane surrounding intracellular *Toxoplasma gondii* funtions as a molecular sieve. Cell Biol..

