# Supplementary material for: ATPe Dynamics in Protozoan Parasites. Adapt or Perish
Source: Genes (Basel). 2018 Dec 27;10(1):16. doi: 10.3390/genes10010016 (PMC6356682; doi:10.3390/genes10010016)
Supplement: Supplementary file 1 [file genes-10-00016-s001.pdf]

```
                   10        20        30        40        50        60        70        80        90       100       110
           ....|....|....|....|....|....|....|....|....|....|....|....|....|....|....|....|....|....|....|....|....|....|....|
TgNTPDase2  LETRCHDSLQALVVIDAGSSSTRTNVFLAKTRSCPNKGIDPDIQLIREGKRFTGLRVVLEEWLDTRPVDARLLFQYVPQMQLLEEDTVAILDSQLNEEQKVQVKALG--I
TgNTPDase1  LETRCHDSLQALVVIDAGSSSTRTNVFLAKTRSCPNKGIDPDIQLIGAGKRFAGLRVVLEEWLDTRPVDARLLFQYVPQMQLLEEDTVAILDSQLNEKQKVQVKALG--I
NcNTPDase   AERRCHDAWQAIVVIDGGSSATRTDVFLAKTRSCPRGGIDPDIRLLGAGKRFAGLRGVLESWLDARSVDSKRLFQHVPEMQLLEDDAVRILDEKLTEEQKVQVQAMG--V
TcNTPDase   SDVGRRQ---TADVFAAIAKEFDEWEDAAQNKTELLTNNAMRRYSIVFDAGSTGSRVHVFRYNLTSLTSFLRLDDELFVEAN-PNDAVASLSPLIEAAKAYIPESLHACV
TbNTPDase   AAVSLQR---AANLIDSLTHQTESWLKKAADTTPSLAAKSPRRYSVVFDAGSTGSRVHVYRYNLTSLLPYLKLEDELFIESD-PRGAAASLVPLLDAAKARVPTDMHACV
LmNTPDase1  VRRMTQQSKRLRVASTLVLSALVIFGFLVYHQSPLFSPSAYAVYDVVIDAGSTGSRVHVFQYERG--RTGFVLLRERFKRATDLDGAKASLEGLLRFADTVVPQSYQKCT
LmNTPDase2  GAVQLRSSSEIRALQAALFHEMRASKKKAENGVASGAAKSSVKYSVVFDIGSTGNRVHVYKYRVASELSDIDLVEELFELENSVQDAPEALWELFVKAKDFVPAELHACT
PfNTPDase   IEGEKKEKKIKERLFLCLICLLVIIWLIYFCYKNHIFLLQNEEYGIIIDAGSNGTRIHLFEWKKRKEIFNAKVKKSISTIDILIYLINKVIDHLIEKKIYVYNKQKWKSY
hNTPDase1   KESNVKTFCSKNILAILGFSSIIAVIALLAVGLTQNKALPENKYGIVLDAGSSHTSLYIYKWPAEQVEECRVKGPGISKFVQKVNEIGIYLTDCMERAREVIPRSQHQET
hNTPDase2   -MAGKVRSLLPPLLLAAAG----LAGLLLLCVPTRDVREPPAKYGIVLDAGSSHTSMFIYKWPADQHSSCDVPGGGISSYADNPSGASQSLVGCLEQALQDVPKERHAGT
hNTPDase3   EQAGLKALYRTPTIIALVVLLVSIVVLVSITVIQIHKQLPPGKYGIVLDAGSSRTTVYVYQWPAEQTFKCSVKGSGISSYGNNPQDVPRAFEECMQKVKGQVPSHLHGST
hNTPDase4   AVSLLYFSVVIIRNKYGRLTRDKKFQRYLARVTDIEATNNPNNYGIVVDCGSSGSRVFVYCWPRHIRQMRDKNRKPVVMKATSPEKVSDYISPLLNFAAEHVPRAKHKET
hNTPDase5   CVCSAVSHRNQQT----WFEGIFLSSMCPINVSAST------LYGIMFDAGSTGTRIHVYTFVQKMPGQLPILEGEVFDSVDQPKQGAETVQGLLEVAKDSIPRSHWKKT
hNTPDase6   YVAYIKWHRATATQAFFSITRAAPGARWGQQAHSPLGTDGHEFYGIMFDAGSTGTRVHVFQFTR-PPRETPTLTHETFKAADDVEKSAQGIRELLDVAKQDIPFDFWKAT
hNTPDase7   SCLLLLMLIIDFRHWSASLPRDRQYERYLARVGELEATEDPNNYGLVVDCGSSGSRIFVYFWPRHIKQMRDRNSQPVVKKADTPEHASDYLRPLLSFAAAHVPVKKHKET
hNTPDase8   MGLSRKEQVFLALLGASGVS--GLTALILLLVEATSVLLPTDKFGIVFDAGSSHTSLFLYQWLANQALACQVEGPGISSYTSNAAQAGESLQGCLEEALVLIPEAQHRKT
DrNTPDase1  MEVKVKNPWHRPVVIFLMAVVAVGIVIMVSISVVQHKPLPQKKYGIVLDAGSSHTSVFIYKWPAEQHHTCNVKGKGISSYFDKPHGAGASLEECMKEAKEKIPAHRHSET
DrNTPDase2  -MVSTGNRQQCQIITATGALFLAVGGILLLVLPSEDIKGSPGMYGIVLDAGSSHTSMFIYKWPADQHSECHAKGGGISSYAGVKGGAARSLKECMDKAMREIPTVRYKHT
DrNTPDase3  SRRGVR-MAKVAVVLAAAFMLASIAVIISIAVVQTNKKVSRGKYGIVLDAGSSRTTVYLYEWPAEQTTKCLVKGPGISDLGDQDQNTWYSLKECMKKMTEAIPSHQHNST
DrNTPDase4  VMGLLYLLLVTGKGQYSWLREDRHIHTRLGRVPDMEATNNPNNYGLVVDCGSSGSRVFVYTWPRHIKQMRDKHRKPVVMKASTPDKASDYISPLLSFAAQHIPKNKHQET
DrNTPDase6  YLTYVKRHYDDSK---LSVPEQLPQYRTDTRKHPTSGQS-ENQYGIMIDAGSTGTRIHIFKFQM-EPNEAPKLAHETFRAADEPEKSKEGILELLNVAKDTVPEVLWSST
DrNTPDase8  -MVHMKAILLGAAMAAVAVT--TIIALILALANRQTLDQPYSQFGIVFDAGSSHTALFLYQWQGNQKQSCDVDGDGISSYVQNPAGAGESLKKCLDIAKAAIPTEQQKST
XtNTPDase5  MIDFSLCHMEPTN----TLKSVLPLATSGQTSHSDT------IYGIMFDAGSTGTRIHIYRFKQRARGSHLELEGEVFESADQPKKGAATVRLLLDLAQKEVPSTQWSHT
XtNTPDase7  VFILVFAAVADLQLWSSRAFRDRQFRRYLDQIEDLEATKDTKNYGVVVDCGSSGSRVFVYFWPPHIKQMRDRGSKPVVKKALTPEKSSDYINPLLSFAASYIPKHKHKET
LpNTPDase   ---MYHSKTSILALYQLMFRVLIFLSCLVILTPNTIADPCEKSCIAVIDAGSTGSRLHIYSYDTDPIHIEEIWNKKIKPGFASIQPNSVTIDAYLTMLLADAP---IHNI
LiNTPDase1  VRRMTQQSKRLRIAGTLVLSALVIFGFLVYYQSPLLSPSAYAVYDVVIDAGSTGSRVHVFQYERG--RTGLVLLRERFKRATDPDGAKESLAGLLRFADKVVPQSYQKCT
LiNTPDase2  GAVELRRSSEIRALQAALFHEMRASKKKAENGVANGEAKTSDKYSVVFDIGSTGNRVHVYKYRVASELSDIDLVEELFELDNPVQDAPEALWELFMKAKYFVPAELHACT
LdNTPDase1  VRRMTQQSKRLRIAGTLVLSALVIFGFLVYYQSPLLSPSAYAVYDVVIDAGSTGSRVHVFQYERG--RTGLVLLRERFKRATDPDGAKESLAGLLRFADKVVPQSYQKCT
LdNTPDase2  GAVELRRSSEIRALQAALLHEMRASKKKAENGVANGEAKTSDKYSVVFDIGSTGNRVHVYKYRVASELSDIDLVEELFELDNPVQDAPEALWELFMKAKYFVPAELHACT

                  120       130       140       150       160       170       180       190       200       210       220
           ....|....|....|....|....|....|....|....|....|....|....|....|....|....|....|....|....|....|....|....|....|....|....|
TgNTPDase2  PVMLCSTAGVRDFHEWYRDALFVLLRHLINNPSYKFFTNWTRPITGAEEGLFAFITLNHLSRRLGCRNDLAGVVEVGGASAQIVFPLQVRAVLQRERLLPERYPSADVVS
TgNTPDase1  PVMLCSTAGVRDFHEWYRDALFVLLRHLINNPSYKFFTNWTRPITGAEEGLFAFITLNHLSRRLGCRNDLAGVVEVGGASAQIVFPLQVRAVLQRERLLPERYPSADVVS
NcNTPDase   PVLLCSTAGVRDFHDWYREALFVILRFLINHPKYKFFTNWTRPITGAEEGLYAFLALNHLSGRLGCRNDLVGVVEVGGASTQIVFPLQIRAVLQHERFLPSRFPCADVIS
TcNTPDase   PIEMKATAGLRRIGREHAEAVLDVVRRLFARGPFWMQSESVRILEGWEEGPLAWLTVNYLLGALNGDKNTATILDLGGGSTQIVMHPSIEAYAEFSYALKVNGRSFVVYQ
TbNTPDase   GIELKATAGLRRIGAEQAGRVLDAVRREFQKGPFWVHSESVRILEGREEGPLAWLTVNFLIGSLSGTKETATILDLGGGSTQVVMRPNLEGHEEFTDTFAINGKSIKLYQ
LmNTPDase1  SVTLKATAGLRLLPESAQQALLDVAQHTLNASPFQSR--GASIISGAQEGVYGWLTVNYLLDRLDTDVATVATIDMGGASTQVVFETTEWLPFNYAYQLRTPKRTIAMYQ
LmNTPDase2  AAEFKATAGLRMLGMEKANEILAGIRARYRNETFWLRGN-VRILDACEEGPMAWLTVNYLLGAFSATDSTVAVIDLGGGSTQIVFEPGHGMRTDFRYAATLGSRSVRAYQ
PfNTPDase   PFYFQATGGMRNLKQEDRNLRMKYIKNILSNDNNPFYFLYARILSGEEEGIYGWLAVNNLLNSIF--NNTYGAIDLGGSSTQITFYPM--HNLENYNSILLNNILIRLYS
hNTPDase1   PVYLGATAGMRLLRMESEELADRVLDVVERSLSNYPFDFGARIITGQEEGAYGWITINYLLGKFSNNQETFGALDLGGASTQVTFVPQQTIEPDNALQFRLYGKDYNVYT
hNTPDase2   PLYLGATAGMRLLNLTNPEASTSVLMAVTHTLTQYPFDFGARILSGQEEGVFGWVTANYLLENFIRK-GTLGAMDLGGASTQITFETTSPAERASEVQLHLYGQHYRVYT
hNTPDase3   PIHLGATAGMRLLRLQNETAANEVLESIQSYFKSQPFDFGAQIISGQEEGVYGWITANYLMGNFLHGVETTGALDLGGASTQISFVAGKMDLTSDIMQVSLYGYVYTLYT
hNTPDase4   PLYILCTAGMRILPESQQKAILEDLLTDIPVHFDFLFSDHAEVISGKQEGVYAWIGINFVLGRFEVRKRTAGILDMGGVSTQIAYEVPNLLAFNLGCDVHQTEHVYRVYV
hNTPDase5   PVVLKATAGLRLLPEHKAKALLFEVKEIFRKSPFLVPKGSVSIMDGSDEGILAWVTVNFLTGQLHHRQETVGTLDLGGASTQITFLPQLEQTRGYLTSFEMFNSTYKLYT
hNTPDase6   PLVLKATAGLRLLPGEKAQKLLQKVKEVFKASPFLVGDDCVSIMNGTDEGVSAWITINFLTGSLKPGGSSVGMLDLGGGSTQIAFLPRLQASPGYLTALRMFNRTYKLYS
hNTPDase7   PLYILCTAGMRLLPERKQLAILADLVKDLPLEFDFLFSQQAEVISGKQEGVYAWIGINFVLGRFDGRRRTVGILDMGGASLQIAYEVPILLAFNLGCDVQHTEHVYRVYV
hNTPDase8   PTFLGATAGMRLLSRKNSSQARDIFAAVTQVLGRSPVDFGAELLAGQAEGAFGWITVNYGLGTLVPEEMLVGALDMGGASTQITFVPGGPILKSTQADFRLYGSDYSVYT
DrNTPDase1  PVYLGATAGMRLLKMEDEMASEKVLTSVAHSLKTYPFSYGARILSGQEEGAFGWITVNYLSENLR--AGTLGALDLGGASTQITFVPQQIIESDNSIDFRLYGNDYHLYT
DrNTPDase2  PVYLGATAGMRLLNISKPKESAEVLKEVEEKLKSYPFSFGASILTGQEEGAYGWITVNYLLENYIGR-DTVGALDFGGASTQITFVTKQTVEKDDLMKLRLYGRDYQIYT
DrNTPDase3  PVFLGATAGMRLLHMTNEGRSNDVLTQMKNYLSSLPFKFNASIISGQEEGLYGWITVNYLKGNFLHGAKTVGSLDLGGASTQIAFATS--DAGEDIIKVSLYGYEYNIYT
DrNTPDase4  PLYILCTAGMRVLPESQQEALLEDLRTDIPVNFNFLFSDHVEVISGKQEGVYAWIGINFVLGRFNLRKRTAGVLDMGGVSTQIAYEVPNLLAFNLGCDAHRTEHVYRVYV
DrNTPDase6  PLMLRATAGLRLLPGEKATILLDKVREVFSESLFLYKPDSVSIMDGTDEGMSAWITINFLIGALHTETPTVGMLDLGGGSTQITFALQIQTSIDYVTSFQMFNISHALYS
DrNTPDase8  PVYLGATAGMRLLNLQNQTQSDSILAEVAKTIQTYPFDFGARILTGMEEGAYGWITTNYLLESFIKAGKIIGALDLGGASTQISFTPKDPVRPNSAFNLQLYGYKYELYT
XtNTPDase5  PVVLKATAGLRLLSETQSDALLSEVREVFHESPFLVPENSVSIINGADEGIFAWITVNFLTGRLHHR--TVGILDLGGGSTQITFLPLLDQTHDFLASVELFNSTYRLYT
XtNTPDase7  PLYILCTAGMRILPESQQIAILQDLVKDVPQEFDFLFSEHAEVISGKQEGVYAWISINFVLGRFDIRKRTVGVIDMGGGSLQIAYEVPSMLAFNLGCDLQHTEHVYRVYV
LpNTPDase   PVYFYATAGMRLLPQSQQKKYYDELEYWFRQQS---QWQEAKTITGNDEALFDWLAVNYKLDTLK--NKSVGVMDMGGASVQIVFPMP-AEIKHNQVELNIYGQNINLYV
LiNTPDase1  FVTLKATAGLRLLPESVQQVLLDAAQHTLNASPFQSR--GASIISGAQEGVYGWLTVNYLLNRLDTDVATVATIDMGGASTQVVFETTEWLPFNYAHQLRTPKRTITMYQ
LiNTPDase2  AVEFKATAGLRMLGMEKATEILDGIRARYRNETFWLRGN-VRILDACEEGPMAWLTVNYLLGVFSATASTVAVIDLGGGSTQIVFEPGHGMRTDLRYSATLGSRSVSAYQ
LdNTPDase1  FVTLKATAGLRLLPESVQQVLLDAAQHTLNASPFQSR--GASIISGAQEGVYGWLTVNYLLNRLDTDVATVATIDMGGASTQVVFETTEWLPFNYAHQLRTPKRTITMYQ
LdNTPDase2  AVEFKATAGLRMLGMEKATEILDGIRARYRNETFWLRGN-VRILDACEEGPMAWLTVNYLLGVFSATASTVAVIDLGGGSTQIVFEPGHGMRTDLRYSATLGSRSVSAYQ

                  230       240       250       260       270       280       290       300       310       320       330
           ....|....|....|....|....|....|....|....|....|....|....|....|....|....|....|....|....|....|....|....|....|....|....|
TgNTPDase2  VSFMQLGMASSAGLFLKELCSNDEVRPDGSASVNEDVRKNPLATYCSVHNFKVTNEMQCRENSITKPLAMKIENCSIIEGTGNVSQVESILVAPKLPLPFESVFRFASST
TgNTPDase1  VSFMQLGMASSAGLFLKELCSNDEVRPDGSASVNEDVRKNPLATYCSVNNFKVTNEMQCRENSITKPLAMKIENCSIIKGTGNVSQVESILVAPKLPLPFESVFRFASST
NcNTPDase   VSFMQLGVASSSGLFFKELCSNAEILPDGTIVVDEDVRKNPVATSCSANNFKAMNEMQCRENKITKSLALRIDDCFQIVGTGDQAQVEELLVSPRFPLPFESVFKFASTA
TcNTPDase   HSYEGNGLHAAKEQLLQAVAANNAVDAFPCFPKGYVHTE-GISN-TRDGGMEECSALFRRHVVRHQPCGSCGFNGVFQPDISAPVYAFSFYYDRLKPY-IKD-IRVQDVL
TbNTPDase   HSYEGNGLNAAREQLLASVATS-PLRAFPCFPQGYAHEK-GVSNGVGGGGMEECVALFRKHVVRGGECSSCGFNGVFQPDVRTPVYAFSFYHDLLRPH-MTAGIRLQDII
LmNTPDase1  HSYLGLGMNEAKKRLMTLFAEANGTLSFPCFPRGYTKRVN---GVELRNSFNACAGLFREHVITTSTCKACGARGVPQPLFPSPIYAFSYFYNRLYDFL--KEVYVSSYK
LmNTPDase2  HSYEGYGLHAATKKLLYHIQGKSAMEAFPCFAVGYEDPLGVKRNNAEEPVFQACANLFRDRLLKGLTCENCGIAGVMQPPLTNEIYAFSFIFDLLVLANVSKEVKLPDLA
PfNTPDase   HSFIGYGWIDSLFRVNIYLCLEIRNYPYNNFQQKYIHTNPNYTKGNINNSKNLSNILLQEIVKYHSQNDYSSLTDIYDYTYNIKPYDIENFIKTIKNFNLVQNPYPYEMK
hNTPDase1   HSFLCYGKDQALWQKLAKDIQVADLYKTPCTK--RFEMTLQQFEIQGIGNYQQCHQSILELFNTCPYS-QCAFNGIFLPPLQGDFGAFSAFYFVMKFLNLTSEVSQEKVT
hNTPDase2   HSFLCYGRDQVLQRLLASALQTHDVYQSPCTMAQRPQNFNARVSLSGSSDPHLCRDLVSGLFSFCPFS-RCSFNGVFQPPVAGNFVAFSAFFYTVDFLRTSMGATLQQLE
hNTPDase3   HSFQCYGRNEAEKKFLAMLLQNSHVFDSLCTVDQRPESYNDVITFEGTGDPSLCKEKVASIFDFCHDQETCSFDGVYQPKIKGPFVAFAGFYYTASALNLSGSFSLDTFN
hNTPDase4   ATFLGFGGNAARQRYEDRIFANTMPYLDPCLPLDIKDEIQQTIYLRGTGDFDLCRETIQPFMNK--NETQTSLNGVYQPPIHFEFYGFSEFYYCTEDVLRMGGYNAAKFT
hNTPDase5   HSYLGFGLKAARLATLGALETE-HTFRSACLPRWLEAEWIVKYQYGGNQEFEPCYAEVLRVVRGHQPEERG------------SFYAFSYYYDRAVDTDDYEKLKVEDFE
hNTPDase6   YSYLGLGLMSARLAILGGVEGQPKELVSPCLSPSFKGEWEVTYRVSGQKAHELCAARVSEVLQNHRTEEHV------------DFYAFSYYYDLAAGVGDAEKLVVGDFE
hNTPDase7   TTFLGFGGNFARQRYEDLVLNETNPFLDPCLPVGLTDVVEQVLHVRGRGDWVSCGAMLSPLLAR--NTSQASLNGIYQSPIDFEFYGFSEFFYCTEDVLRIGGYHGPTFA
hNTPDase8   HSYLCFGRDQMLSRLLVGLVQ-SPLYESPCVHATPP--LSQNLTVEGTGNPGACVSAIRELFNFCQGQEDCAFDGVYQPPLRGQFYAFSNFYYTFHFLNLTS-QPLSTVN
DrNTPDase1  HSFLCYGKDQALKLAMAEKLRSTSVN-TPCMK--PLKMPKEQFSHVGLGNWSQCQESIRKVFNTCPYS-GCSFNGVFQPTVEGKFGAFSAFFFVMDFLNLKND-SLDKTK
DrNTPDase2  HSFLCYGRDQVLLRLLAHLITIQSVFGTPCVEKQTS--YNGELQIKGTGNYNQCLGNVSQLFSFCSYS-KCSFDGVFQPNITGNFMAFSAFFYTHSFLQEATGWTPAHLE
DrNTPDase3  HSFLCYGKNEAEKRVLAKLAKEPEIFGSECTKNNVPSRYNRLITFFGQSNPEQCKALVRSIFDLCQGTENCSFDGVYQPPLNGDFTAYAGFYWTALALKVNGSNSMEKFN
DrNTPDase4  STFLGFGGNAARQRYEENLISSTSPILDPCLPVDLQDELGQRLHLRGSGDFERCRLLLQPFLNR--NDTNTSLNGVYQPPIDFQFYGFSEFYYCTEDVLRMGGYNSTKYT
DrNTPDase6  HSYLGLGLMSARLAVLGGIEGQPQELVSPCLAPDYSGQWEVLYTVKGQKPYESCLRKVEKMIYKRKAEENM------------DFYAFSYYYDRAVDLGDERTVRVSDYI
DrNTPDase8  YSYLCYGKDQALKKLQAYLHKNAELYNSPCVVPPSS--FNDTILFKGTGNSSLCLSLIENIVNLCALSPDCGFNGIYQPPVNGEFFAFSAYFYTFDFLGLTP-APLTKVL
XtNTPDase5  HSYLGLGLKLARLAVLGVSEDN-QFFHSNCFLHGVKGEWSVTLKYEKKSDFDACYSEILPIIQDHQVPEHS------------PFYAFSYYYDRAADANDHMHLQVYDFA
XtNTPDase7  TTFMGFGGNFARQRYEDMVFNDTSPLQDPCLPVGLIDQVRHDLHVLGKGNWDSCRQQLEPLLLK--NDTQAYLNSVYQPSIDFEFYGFSEFFYCTEDVLRMGGYNSQKFA
LpNTPDase   HSFLGLGQTEMSHQFLN---------SPSCFANDYPLPDG------GQGNAPSCKEEVTSLMNSKVNQQPLLALNPVNEWYSINLASSQLFHFENSELT-----NQSLLQ
LiNTPDase1  HSYLGLGMNEAKKKLMMSFAEANGTSSFPCFPRGYAKRLN---DVELRNSFDACAGLFREHVITKTTCKACGARGVPQPLFPSLIYAFSYFYDRLYHFS--KEVYVSSYK
LiNTPDase2  HSYEGYGLHAATKELLFHIQGKSAVEAFPCFAVGYEDPLGIKKNNTGEPAFQACANLFRDRLLKGLTCENCGIAGVMQPPLTNEIYVFSFIFDLLALANVSREVKLPDLA
LdNTPDase1  HSYLGLGMNEAKKKLMMSFAEANGTSSFPCFPRGYAKRLN---DVELRNSFDACAGLFHEHVITKTTCKACGARGVPQPLFPSLIYAFSYFYDRLYHFS--KEVYVSSYK
LdNTPDase2  HSYEGYGLHAATKELLFHIQGKSAVEAFPCFAVGYEDPLGIKKNNAGEPAFQACANLFRDRLLKGLTCENCGIAGVMQPPLTNEIYVFSFIFDLLALANVSREVKLPDLA

                  340       350       360       370       380       390       400
           ....|....|....|....|....|....|....|....|....|....|....|....|....|..
TgNTPDase2  APMFITGREMLASIDTLKDEELAEAAREFCSSEVIIRTDGPVLPNARGEQKLNSLDLCKTMALTVSLLRHMA
TgNTPDase1  APMIVTGGGMLAAINTLKDEELAEAAREFCSSEVIIRTDGPVLPNARGEQKLNSLDLCKTMALTVSLLRHMA
NcNTPDase   SPMVITGGAMYASISTMQGEQLIAASRTYCSSPVVNSGDGLVLPNA--EQKLTSMDLCKTIALTVSLIQHME
TcNTPDase   DIASQVCRSMKSVRELLEQPEMECFELSYLFTLLRYGFGFPPEQKLHIAKKINGFETAWALGASLVSLEGQS
TbNTPDase   GIASQVCKSMKSVEQLRREPELECLHLSYVVALLRDGFHYPLDQELHIAKKIDGYETAWTLGASLVSLEEHC
```

```
LmNTPDase1  EVGQEVCHRESARRTTAP-EETACMELAYMYSFLTYGLGLSDATVLTVPNRIEGMAVSWSLGSSLSFLLKME
LmNTPDase2  KIAEGHCAAFSLTRIAEATPEYECMYYSYVYALLRYGYEVPEDRVLHVAKKIRGYETAWSLGASLLSLT---
PfNTPDase   ITFPTYNLVTSQFVILKKNNNYTQNDGNYYRQNVGNYYRHNHNDVYELNSSESYDISNFVLKLKIINNNMSL
hNTPDase1   EMMKKFCAQPWEEIKTSYALSEYCFSGTYILSLLLQGYHFTAWEHIHFIGKIQGSDAGWTLGYMLNLTNMIP
hNTPDase2   AAAVNVCNQTWAQLQARVPLADYCAGAMFVQQLLSRGYGFDEFGGVIFQKKAADTAVGWALGYMLNLTNLIP
hNTPDase3   SSTWNFCSQNWSQLPLLLPARSYCFSANYIYHLFVNGYKFTEWPQIHFEKEVGNSSIAWSLGYMLSLTNQIP
hNTPDase4   KAAKDYCATKWSILRERFDLKYQCFKSAWMFEVFHRGFSFPVYKSLKTALQVYDKEVQWTLGAILYRTRFLP
hNTPDase5   RKAREVCDNLENF-TSGSP--FLCMDLSYITALLKDGFGFADSTVLQLTKKVNNIETGWALGATFHLLQSLG
hNTPDase6   IAAKYVCRTLETQ-PQSSP--FSCMDLTYVSLLLQE-FGFPRSKVLKLTRKIDNVETSWALGAIFHYIDSLN
hNTPDase7   KAAQDYCGMAWSVLTQRFKLKYQCFKSAWMYQVLHEGFHFPYYPNLRTAQLVYDREVQWTLGAILYKTRFLP
hNTPDase8   ATIWEFCQRPWKLVEASYPLRDYCASGLYILTLLHEGYGFSEWPSLEFRKQAGGVDIGWTLGYMLNLTGMIP
DrNTPDase1  QRLAMYCSTPWQKIVQDHPLSEYCFSATYILTLLEHGYNFTSWNDIKFIKKIGDSDAGWTLGYMLNLTNMIP
DrNTPDase2  DAARVICNMSLQEMGKKSQLKDYCAVSVFVRALLVNGYSFNDFPQISFQKKAGDTSVGWSLGYMLSLSNLLP
DrNTPDase3  TNMKLFCSNDWKNLKQNYELKSFCYSANYVHTILADGYKFNTWENLEFSKEVNNTSIAWSLGYMLALSNMIP
DrNTPDase4  NAAKSYCATQWKTLKERFDLKYQCFKSAWMHEVLHSGFAFPAYENLRTALLVYDKEVQWTLGAILYRTRFLP
DrNTPDase6  EGAKKVCNNMSAGGIKESP--FLCLDLTYISVLLQE-LDFPPDKELKLARQINNAETSWALGAAFHCIESFR
DrNTPDase8  STIDTLCSKNWTALTAENPLKDYCASGHYITTILLKGYKFNNWDQISFVKQVADTDIGWTLGYMLNLTNMIP
XtNTPDase5  KSAKEVCERTEQS-PTHSH--FLCMDLTFITALLKEGFGFEDNTSLQLTKKMYDVEMSWTLGAIFHVLQSLH
XtNTPDase7  KAAKEYCSMPWTTLQDRFNLKYQCFKSAWMFSILHNGFHFPHYPNFKTAQLVYDKEVQWTLGAILYKTRFLP
LpNTPDase   QGDNQICHQQWDILNGQYPLYQYCLLSSYYYALMVDGYGINPNQTIHYIPPEQNL--DWTIGVVLHRA----
LiNTPDase1  EVGQEVCHRESARRTTAP-EETACMELAYMYSFLTYGLGLSDATALTVPNRIEGMAVSWSLGSSLSFLLKME
LiNTPDase2  TIAEGHCAAFSLTRIAEATPEYECMYYSYVYALLRYGYEVPEDRVLHVVKKIRGYETAWSLGASLLSLT---
LdNTPDase1  EVGQEVCHRESARRTTAP-EETACMELAYMYSFLTYGLGLSDATALTVPNRIEGMAVSWSLGSSLSFLLKME
LdNTPDase2  TIAEGHCAAFSLTRIAEATPEYECMYYSYVYALLRYGYEVPEDRVLHVVKKIRGYETAWSLGASLLSLT---
```

Figure S1. Sequence alignment of NTPDases used to build the evolutionary tree of Figure 2. TrimAl software was used to delete sites exhibiting a high proportion of gaps(GAPPYOUT function). NTPDases and their accession numbers are listed in Figure 2.
